# Fluorescence of various buried fresh and fresh-frozen-thawed tissue types up until the point of active decay: a human taphonomy study

**DOI:** 10.1007/s00414-024-03387-w

**Published:** 2024-12-20

**Authors:** Emmanuelle Charlot, Anas Gasser, Roelof-Jan Oostra, Maurice C. G. Aalders, Tristan Krap

**Affiliations:** 1 Department of Medical Biology, Section Clinical Anatomy and Embryology, AmsterdamUMC, Location Academic Medical Centre, Meibergdreef 9, Amsterdam, AZ 1105 The Netherlands; 2 Department of Biomedical Engineering and Physics, Amsterdam UMC, Location Location Academic Medical Centre, Meibergdreef 9, PO Box 22700, Amsterdam, DE 1100 The Netherlands; 3 Co van Ledden Hulsebosch Center, Science Park– Building 904, (Room C2.243), Amsterdam, XH 1098 The Netherlands; 4 Faculty of Law and Criminology, Maastricht University, Minderbroedersberg 4–6, Maastricht, LK 6211 The Netherlands

**Keywords:** Forensic taphonomy, Freeze-thaw cycle, Fluorescence spectroscopy, Protein-lipid oxidation

## Abstract

Forensic taphonomy is the study of postmortem changes of human remains for the purpose of answering legal investigative questions. Many variables can affect the pattern and rate of decomposition of remains, posing challenges for taphonomic studies and estimation of the postmortem interval. Given the gap in knowledge regarding the suitability of using frozen remains to extrapolate conclusions to fresh material, investigating the effects of freeze-thaw cycles followed by burial on human remains is vital for forensic practice and taphonomic research. This study explored the impact of a freeze-thaw cycle and subsequent burial on human tissue decomposition under semi-controlled field conditions. Fresh and fresh-frozen-thawed hands were buried at the Amsterdam Research Initiative for Sub-surface Taphonomy and Anthropology for 31.7 to 340.4 accumulated degree days. Decomposition was assessed using fluorescence measurements targeting protein and fluorescent oxidation products, and broader excitation-emission matrix measurements in skin, adipose, and muscle tissue. Decomposition trends varied primarily by treatment group: fresh samples generally aligned with expectations that protein levels would decrease over time while fluorescent oxidation products increased, whereas fresh-frozen samples deviated significantly from these expectations. Significant differences were found between protein and fluorescent oxidation products levels of fresh and fresh-frozen tissue at corresponding time points, indicating this method’s potential in determining sample state. However, fluorophore peak monitoring in excitation-emission matrices did not prove useful in establishing decomposition trends or potentially distinguishing between sample states. Despite limitations inherent to pilot and human taphonomy studies, this study clearly demonstrates that differences exist in the decomposition of fresh and fresh-frozen tissue, and that these trends vary slightly by tissue type. We therefore conclude that frozen material cannot be considered a proper substitute for fresh tissue regarding taphonomic processes, and the methods used in this study show promise in being used to differentiate between pre-decomposition treatments.

**Supplementary Information:**

The online version contains supplementary material available at 10.1007/s00414-024-03387-w.

## Introduction

Forensic taphonomy is the study of decomposition processes and the factors that influence them, with the aim of being applied to matters of the law [1, 2]. Due to the wide array of variables which affect this process, the study of taphonomy and evaluation of the postmortem interval (PMI) are complex tasks requiring a strong foundation of knowledge regarding how, and to what extent, these variables affect the pattern and rate of decomposition of remains. Thus, studies targeting variables suspected to influence decomposition are instrumental in expanding the body of knowledge of forensic taphonomy. Though the number of taphonomic studies undertaken in recent years continues to grow, very few of these have explored the effects of freezing on decomposition, much less those of freezing followed by burial. To our knowledge, only one published work addresses the taphonomy of frozen and subsequently buried tissue using a non-human substitute for human tissue [2].

Decomposition refers to the biochemical processes that organic matter undergoes after death, and is made up of three main stages: autolysis, putrefaction, and decay [3]. When a cadaver is buried, the stages of decomposition remain the same, however, the rate at which these occur differ compared to an exposed body [3]. Decomposition, especially decay, tends to be slowed by burial due to decreased insect and scavenger activity, decreased temperature, and soil properties affecting pH, moisture, and available oxygen [3, 4]. The research by Stokes, Forbes & Tibbett [2] is, to our knowledge, the only study which has addressed the effects of freezing and burial on decomposition. The authors buried sections of frozen-thawed or refrigerated *Sus scrofa* muscle in sand microcosms for up to 37 days, and measured decomposition as a function of tissue mass loss and carbon dioxide respiration [2]. No significant differences in these parameters were observed between treatments, however, histological analysis revealed a substantial increase in interstitial space size in frozen tissue [2]. One unpublished study on the decomposition of frozen-thawed-buried and fresh-buried pig legs over a 60 day period revealed that fresh limbs decomposed faster than frozen limbs overall (T. Krap, personal communication, August 30 2022). A handful of studies on the decomposition of exposed frozen cadavers have been undertaken and suggest that freezing tends to lead to slower decomposition, especially during early decomposition, and decomposition from the outside-in [1, 5].

Fluorescence spectroscopy is a valuable tool which can be used to provide insight into the biochemical changes that occur during tissue decomposition. This method has previously been utilized in the food industry to discriminate frozen-thawed from fresh meat, based on the spectrochemical profiles measured at given excitation wavelengths, with high accuracy in discriminating between such samples [6, 7]. Fluorescence occurs when a sample emits light after having absorbed light [8]. Fluorophores are molecules with fluorescent properties [9]. Excitation and emission wavelengths differ between fluorophores [8]. Endogenous fluorophores in human skin, adipose, and muscle include collagen, lipids, and elastin (see Table 1 for a detailed overview of endogenous fluorophores) [10–15]. In fluorescence spectroscopy, the electromagnetic radiation emitted from a sample is detected and measured, and can be used to identify a molecule or as an indication of changes within a molecule [16]. Degradation mechanisms of various forensic traces such as fingermarks and body fluids have been studied by monitoring proteins (PROTs) and their oxidation to form fluorescent oxidation products (FOX) via fluorescence spectroscopy and excitation emission matrices (EEMs) [17–19]. EEM plots display the fluorescence of a sample against various emission and excitation wavelengths, and can therefore be used to visualize the locations of specific fluorophores, which are visible as regions of high intensity fluorescence [17]. It is assumed that, upon contact with air, unsaturated fatty acids will oxidize to form reactive oxidation products [17]. These in turn react with PROTs to form FOX, with the fluorescence intensity of FOX assumed to be directly proportional to its quantity [17]. Compounds hypothesized to contribute to PROT fluorescence are elastin and tryptophan, while contributors to FOX fluorescence include various tryptophan derivatives (kynurenine and protein carbonyls), and NADH [10–12, 14, 15, 17, 20–23]. Several studies using non-human tissue have reported a decrease in PROT fluorescence, and simultaneous increase in free fatty acids and protein carbonyls as the number of freeze-thaw (F-T) cycles increases [24–26]. PROT and FOX content therefore appears to fluctuate over time, and depending on sample state. Based on the available literature concerning the ageing mechanisms of PROT and FOX, it is expected that the PROT emission peak will decrease over time, while that of FOX increases [17–19]. Hence, the PROT-FOX ratio is expected to decrease over time [17–19]. When whole hands are buried, the dermal layer is most exposed to the soil and available oxygen present therein, while adipose is shielded by the skin, and muscle is itself shielded by adipose and skin. Patterns in the occurrence and levels of PROT and FOX are expected to differ between fresh (F) and fresh-frozen (FFR) tissue, perhaps displaying more significant changes in FFR samples, and with skin exhibiting a more rapid oxidation of PROT to FOX.

This study not only explored the effects of freezing and subsequent burial on human tissue, but more notably, under semi-controlled conditions, wherein severed human hands were exposed to a burial environment with little variation in composition. Furthermore, precipitation and temperature were monitored, control tissue samples for each treatment group were used to establish baseline measurements, and sampling and measurement protocols were consistent across treatment groups. This was made possible by the use of the Amsterdam Research Initiative for Sub-surface Taphonomy and Anthropology (ARISTA) [27]. Furthermore, the use of various fluorescence spectroscopy measurements, an increasingly prevalent forensic analytical method, ensured that differences in the taphonomy of F-buried and FFR-buried tissue were captured in more than one manner. This topic is crucial for future taphonomic research if frozen material is found to decompose differently from fresh tissue, as frozen material may no longer be suitable in extrapolating conclusions to fresh material. This could change our current understanding of human taphonomy. In addition, such research may help to shed light on the *modus operandi* of a perpetrator, and allow investigators to construct a more accurate timeline of events for a crime, thereby benefitting the overall criminal investigation. The goals of this study were to investigate.

- to what extent F and FFR human tissue can be discriminated based on fluorophore peak monitoring in EEMs and PROT-FOX fluorescence measurements,
- and to what extent trends in decomposition vary between the F and FFR groups for the same tissue type, based on fluorophore peak monitoring in EEMs and PROT-FOX fluorescence measurements.

In addition, the effect of multiple F-T cycles was explored upon re-freezing of samples for one time point in the FFR group.

## Materials & methods

### Hand donors and burial site

Twenty-two hands, obtained through the Department of Medical Biology’s body donation program at Amsterdam UMC (location AMC) were buried at ARISTA (see the compliance with ethical standards section below for details concerning the use of human tissue for scientific research) [27]. Eleven donors contributed to this experiment, with 16 FFR hands paired off by body donor for a total of eight FFR pairs, likewise for the six F hands for three F pairs. Donors with characteristics affecting decomposition (i.e. low/high body fat, and signs of infection/necrosis) were excluded. The male to female donor ratio was 1.75 (7:4). See electronic supplementary material (ESM) A for an overview of donor characteristics.

The burial site, a 130 cm x 130 cm area of ground at ARISTA’s southern edge, consisted of a top 10 cm layer of humus and sand, and a deeper 10 cm layer of, primarily, sand. On September 20th 2022, 16 burial pits measuring 10 cm in length and width, 20 cm in depth, and with 20 cm of spacing between each pit, were dug. FFR hands were thawed at room temperature for 6 h before burial, except control hands, which thawed for 4.5 h prior to sampling. This difference in thawing times arose from the varying time required for preparing samples for burial and sampling. Pits of the same dimensions and spacing were dug for each F hand on their respective burial days. Hands, with the most distal part (fingers) pointing downward, and the most proximal part (point of severance) upward, were buried in no particular order. The excavated soil was replaced without regard to original soil layer depths, so as to mimic the actions of an individual disposing of human remains. Each burial pit was marked with a plastic label at the center of the pit.

### Experimental design

#### Temperature, precipitation, and qualitative observations of decomposition

A temperature probe was used to record the temperature of the air and soil at the burial site at a depth of 20 cm, and the internal temperature of each hand upon burial, and exhumation. Precipitation was monitored via a nearby weather station [28]. To ensure consistent exposure of hands to water across treatment groups, burial pits were watered with clean tap water in case of prolonged drought (see ESM B).

Qualitative observations of decomposition were made upon each exhumation, and monitored variations in hand coloration, signs of livor mortis, skin slippage, marbling, bloating, and any other notable changes.

#### Exhumations and sampling

One pair of control hands from each treatment group were used to ascertain baseline measurements. Exhumations took place on days 2, 4, 6, 9, 14, 20, and 24 for FFR hands, and at almost equivalent accumulated degree days (ADD) to that of FFR days 14 and 24 for F hands.

On each exhumation day, a pair of hands from the FFR or F group was exhumed based on the exhumation scheme (see ESM C), and temperature measurements, and photographs were taken. At the morgue, hands were washed under clean tap water, and new photographs were taken prior to sampling. Two biopsies containing skin, adipose, and muscle tissues, were subsequently taken from the palmar side of each hand, individually placed in tissue cassettes, and stored in a 600 mL Duran bottle containing 500 mL 10% neutral buffered formalin for future histological research.

Tissue samples to be analyzed by fluorescence spectroscopy were taken from FFR time points 0, 2, 6, 14, and 24 days hands, and all F hands. A 3 cm by 2 cm section of skin and adipose was excised over the second metacarpal of each hand, then cut in two, resulting in two samples per hand. A 3 cm cut was made over the first dorsal interosseous muscle, and two pieces of muscle were extracted from each hand. An illustration of the sampling areas is provided in ESM D. Samples were individually placed in labelled tissue cassettes, and refrigerated at 4 ºC until analyses could be performed.

FFR time point 2 samples were frozen after initial measurements were taken. As samples from later time points necessitated different LS55 settings, these time point 2 samples were re-measured to ensure consistency. Consequently, FFR time point 2 samples were used to assess the effect of one vs. two F-T cycles on PROT and FOX fluorescence.

#### Fluorescence spectroscopy

The optical characteristics of skin, adipose, and muscle tissue were assessed using a PerkinElmer LS55 fluorescence spectrometer. Tissue samples were transferred into labelled Cellstar^®^ dishes, and positioned so that the sample lay 0.5 cm below the spectrometer’s excitation-emission fiber bundle. Optimized settings used to analyze each tissue type are detailed in ESM E. PROT and FOX fluorescence spectra were measured in triplicate for each sample at various locations, and one EEM measurement per tissue type at each time point for each treatment group was taken (see ESM E for LS55 settings). In case of saturated peaks, a neutral density (ND) filter with optical density 0.1 was used. A detailed procedure for this method is given in ESM F.

### Data analysis

#### Fluorescence spectroscopy

PROT-FOX and EEM measurements were processed using a custom MATLAB^®^ (Version R2022b) app. Thresholds for calculating PROT and FOX area under the curves (AUCs) were established by plotting the measurements in Microsoft Excel. Wavelength ranges capturing the emission maxima and adjacent regions for the largest number of measurements for time points 0 and 24 days were determined within each tissue type and treatment group combination. These ranges were then used to obtain the threshold which would capture the emission maxima and adjacent regions for the majority of measurements within each tissue type. Refer to ESM G for an example of this method and an overview of the thresholds set.

PROT and FOX AUCs, and the PROT-FOX ratio, were calculated from each triplicate sample measurement. AUCs were calculated using the ‘trapz’ function in MATLAB^®^. The ratio of the PROT AUC peak to the FOX AUC peak was then calculated. This resulted in three PROT AUCs, FOX AUCs, and PROT-FOX ratios per sample. In instances where an ND filter was used, measurements were scaled prior to AUC and ratio calculations according to the procedure described in ESM F.

**Fig. 1:**
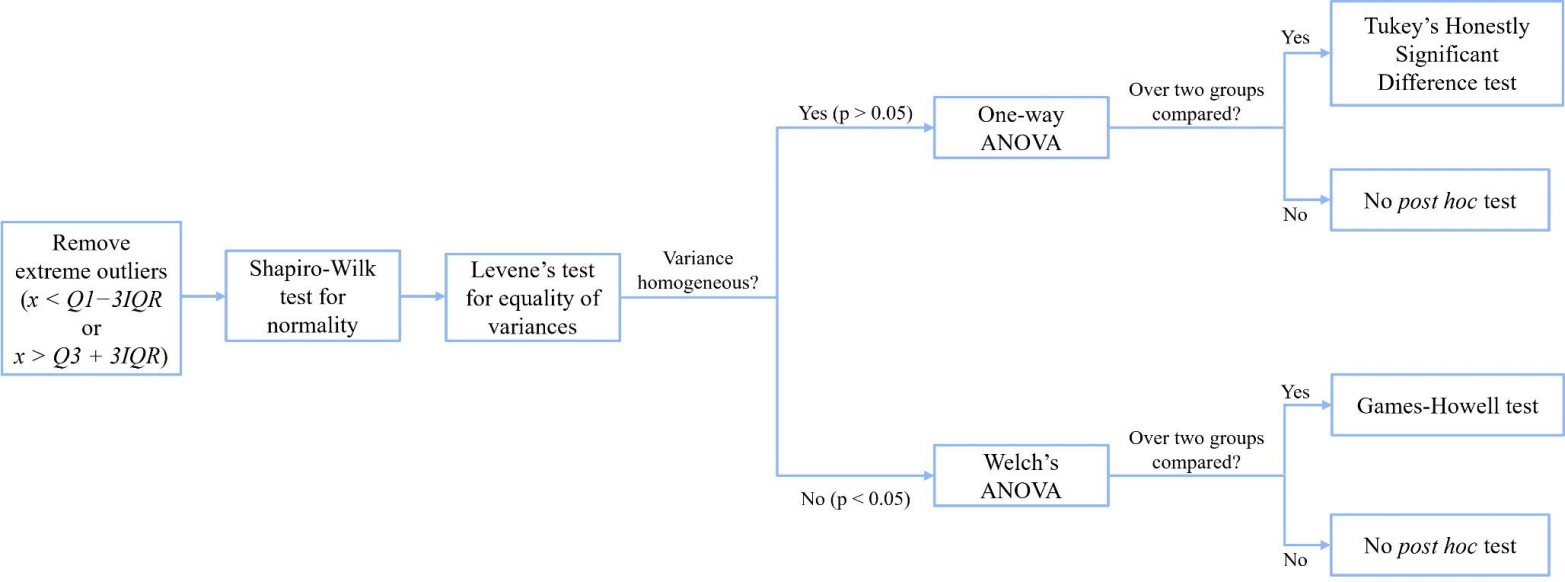
Workflow for statistical analysis of PROT and FOX AUCs and ratios in SPSS®

AUC values and ratios were analyzed over time per tissue type/treatment group, and between treatment groups per tissue type, using SPSS^®^ following the workflow described in Fig. 1.

Scatter plots for AUCs and PROT-FOX ratios were constructed for each tissue type in SPSS^®^ after removal of extreme outliers. Trend lines were fitted to the F and FFR datasets, with the line of best fit deemed to have been found for the trend line type which yielded the highest R^2^ values for both datasets concurrently. It was however ensured that the chosen trend line was not overly complex: if a higher R^2^ had been achieved using a more complex model, a simpler trend line would have been chosen to avoid overfitting and maintain interpretability. An example of the statistical procedure can be found in ESM H.

Prior to 2D rendering of EEMs, a cleaning function in MATLAB^®^ was used to remove excitation light and 2nd order scattering, each dataset was then normalized to its highest peak. Matrices were then rendered as 2D contour plots using the ‘contourf’ function. Fluorophore peaks were identified, and their presence/absence and fluorescence intensities were monitored in each EEM to assess their changes over time, and between treatment groups. A description of this method is detailed in ESM I. A preliminary literature search was conducted to establish optimal excitation and emission wavelengths for the endogenous fluorophores expected to contribute to tissue fluorescence. An overview of these is shown in Table 1.

**Table 1:** Overview of endogenous fluorophores expected to be observed in EEMs

Endogenous fluorophore	Optimal excitation/emission wavelengths (nm)
Bilirubin	(470/520–540 [29]) (464/540 [30]) (474–480/530 [31])
Collagen	(340/390 [11]) (345/395 [12]) (337/380–420 [13])
Elastin	(285/350 [15]) (290/365 [16])
Flavins	(455/495 [15]) (460/550 [11]) (440–450/540 [14]) (440–450/500–550 [32])
Kynurenine	(380/480 [33]) (365/480 [34]) (360/460–470 [35])
Lipids	(330–350/470–480 [14])
Melanin	(800/500 [36]) (760/455 [37])
NADH	(351/460 [13]) (355/475 [11]) (350/470 [12])
NADPH	(336/464 [13]) (340/450 [12]) (340/465 [38])
Oxyhemoglobin	(480/420, 540, 580 [39]) (476/420, 540, 580 [40])
Protein carbonyls	(360/385 [41]) (350/435 [42]) (350/450 [43]) (350/400–500 [23])
Porphyrins	(405/610 [15]) (405/600 [24]) (405/600 [13]) (400/630 [11]) (405/630 [12])
Tryptophan	(295/340–350 [24]) (295, 280/345, 350 [13]) (260–295/325–375 [18])
Tyrosine	(280/312 [13]) (270/320 [44]) (275/300 [12])

Time point 2 FFR samples were re-measured following re-freezing after initial measurements, as LS55 settings had to be altered to analyze samples at later time points. This was taken as an opportunity to investigate the effects of multiple F-T cycles on PROT-FOX fluorescence. The ratio of PROT and FOX fluorescence prior to, and after, a second F-T cycle was calculated for each measurement type respectively by dividing the fluorescence intensity at each emission wavelength after one F-T cycle, by the values obtained after the second cycle. These were then graphed to compare the behavior of both fluorophores.

## Results

### Deviations in experimental procedure

On December 2nd 2022, both pairs of F hands were exhumed *en bloc* in their original taphonomic contexts, and individually placed in clean plastic bins, as below-freezing temperatures were expected. Initially, bins were stored in the ARISTA shed, and were later transferred to the AMC morgue on December 8th to reach their target ADD due to the increasingly cold weather (outdoor air temperature of 4.4 °C vs. indoor shed temperature at 5 °C on 08/12, average outdoor air temperature of − 0.2 °C on 09/12).

Scavenging was discovered on December 2nd 2022, with the F time point 14 left hand partially exposed, and the burial pit of the right hand disturbed without the hand being exposed. No damage to the hands was observed, and the pits were promptly refilled upon discovery. Scavenging likely occurred within 4 days of its discovery, as the heavy rainfall prior would have otherwise washed away the thick layer of soil present on the exposed hand.

### Donor characteristics

No significant difference in age between male and female donors was found (*p* > 0.05, ANOVA).

### Temperature, precipitation, and qualitative observations of decomposition

Air and soil temperatures were compared during each FFR exhumation to assess discrepancies between these. Variations not exceeding 1 °C were observed between air and soil at a depth of 20 cm. Therefore, air temperature was deemed suitable in calculating ADD, with a threshold of 0 °C, as below this temperature enzymatic and bacterial activity, and therefore decomposition, are inhibited [45, 46]. In instances where air temperature data was unavailable, data from a nearby weather station were used [28]. Table 2 details ADD for each hand pair. The discrepancy in ADD between F and FFR hands at time points 14 and 24 days reflect temperature differences during the burial and exhumation periods of late September to mid-October for FFR hands, and early November to mid-December for F hands.

**Table 2:** ADD for each hand pair

	FFR hand pairs								F hand pairs		
ADD	0	31.7	58.7	82.8	115.6	179.9	257.6	300.4	0	196.9	340.4
Time point (days)	0	2	4	6	9	14	20	24	0	14	24

At time point 24 days, FFR hands were exposed to 6.5 mm less total precipitation (including watering) than the corresponding F pair, while at time point 14, F hands were exposed to 26.7 mm less total precipitation than the corresponding FFR pair. Pits for FFR hands were not left to dry for more than 24 consecutive hours, while F hands were not left to dry for more than 48 h at a time, with one exception where drying lasted for 72 h. These discrepancies reflect the challenge in maintaining consistency in moisture exposure across treatment groups due to the unpredictable nature of weather and inaccuracy of forecasts. Details on precipitation have been compiled in ESM J.

Hand discoloration was observed at all time points for both FFR and F hands. At time point 14 days, FFR hands had begun turning green, with extensive slippage and some brown discoloration at the severance points. By contrast, F hands at the same time point were greyish-pink with extensive skin slippage but no brown discoloration. At time point 24 days, FFR hands displayed extensive skin slippage and further brown discoloration, while F hands showed some brown discoloration, almost complete fingernail loss on the right hand, and loosely attached fingernails on the left hand, loose thumbs due to tissue mass loss, and blue tissue on the dorsal side of the left hand, likely due to a fungus. Refer to ESM K for detailed visual observations.

### Fluorescence spectroscopy

Fluorescence signals in the wavelength range for PROT were saturated in all F skin and one F muscle sample at time point 0, and one F muscle sample at time point 14 days. An ND filter was used to measure these samples again. Three extreme outliers from the adipose dataset, one from the muscle dataset, and two from the skin dataset were removed prior to statistical analysis. An overview of these outliers is shown in ESM L.

#### Decomposition over time by tissue type and treatment

Relationship significance between time points for each tissue type within each treatment group, and corresponding scatter plots with lines of best fit are summarized ESM M. The best fit (highest R² possible for both treatment groups concurrently) trend line was obtained when applying a quadratic function to the datasets. R² and quadratic coefficient values are displayed in Table 3.

**Table 3:** R² and quadratic coefficient sign (+ or −) for each treatment group, by parameter and tissue type

Tissue type	Parameter	Treatment group	*R*²	Quadratic coefficient sign (+ or −)
Skin	PROT AUC	FFR	0.01	−
		F	0.93	+
	FOX AUC	FFR	0.27	+
		F	0.58	+
	PROT-FOX ratio	FFR	0.12	−
		F	0.93	+
Adipose	PROT AUC	FFR	0.42	+
		F	0.95	+
	FOX AUC	FFR	0.43	+
		F	0.53	+
	PROT-FOX ratio	FFR	0.06	−
		F	0.76	+
Muscle	PROT AUC	FFR	0.38	+
		F	0.66	−
	FOX AUC	FFR	0.08	−
		F	0.86	+
	PROT-FOX ratio	FFR	0.10	+
		F	0.57	−

##### Skin

PROT-FOX ratios also did not yield a clear significance pattern over time in FFR skin, while all comparisons in the F group resulted in significant differences. A trend line with a negative quadratic coefficient provided a poor fit for the FFR dataset. PROT-FOX ratios increased variably until time point 6 in FFR skin, followed by a decrease at time point 14, and remained relatively unchanged at time point 24 compared to time point 14. PROT-FOX ratios decreased over time in the F group, while a trend line with a positive quadratic coefficient resulted in a very good fit for this group.

##### Adipose

The ANOVA test produced significant differences between PROT AUCs for every time point comparison in adipose, except for the time point 0 to time point 6 comparison in the FFR group, and time points 14 to 24 in the F group. Quadratic trend lines displayed a moderate fit for the FFR dataset, and very good fit for the F dataset. In both instances, the quadratic coefficient was positive, with PROT declining over time in both groups.

Significant differences in FOX AUCs emerge from time point 14 in FFR adipose, and from time point 24 in F adipose. Trend lines with positive quadratic coefficients provided a moderate fit for each treatment group. An initial decrease in FOX was observed in both treatment groups, and was more marked in the FFR group, followed by an increase.

PROT-FOX ratios did not display a clear significance pattern in FFR adipose, but significant differences were present at all time point comparisons in the F group. A trend line with a positive quadratic coefficient provided a good fit for the F dataset, while the trend line for the FFR group showed a very poor fit and a negative quadratic coefficient. Similarly to F skin, PROT-FOX ratios decreased over time in F adipose, and displayed a slight increase until time point 14 in FFR samples, followed by a decrease at time point 24.

##### Muscle

Only comparisons to time point 24 produced significant differences between PROT AUCs in FFR muscle, while all comparisons in the F group yielded significant differences. A trend line with a positive quadratic coefficient produced a poor fit for the FFR dataset, and a trend line with a negative quadratic coefficient resulted in a moderate fit for the F dataset. PROT levels appeared to decrease until time point 14 in FFR muscle, followed by an increase, while in F samples, a decrease over time was observed.

FOX AUCs showed no clear pattern of significance over time in FFR muscle, but significant differences were present for comparisons to time point 24 in F samples. A trend line with a negative quadratic coefficient resulted in a very poor fit for the FFR dataset, while a trend line with a positive quadratic coefficient produced a good fit for the F dataset. Similarly to F adipose, FOX levels in F muscle displayed an initial decrease followed by an increase. On the other hand, FOX displayed a slight increase until time point 6, followed by a decrease in FFR samples.

PROT-FOX ratios displayed an inconsistent pattern of significance over time in FFR muscle, while significant differences emerged from time point 24 in F muscle. A trend line with a positive quadratic coefficient provided a very poor fit for FFR PROT-FOX ratios, which displayed a slight increase over time. A trend line with a negative quadratic coefficient yielded a moderate fit for the F dataset, with ratio values significantly decreasing at time point 24.

#### Fresh vs. fresh-frozen

ANOVAs and corresponding *post hoc* tests were conducted to identify significant differences between F and FFR samples at corresponding time points for the same tissue type. Significant differences between treatment groups were found for at least two parameters (PROT AUCs, FOX AUCs and/or PROT-FOX ratios) across tissue types, except for the comparison at time point 14 in skin samples, where no significant difference was found. Only one non-significant relationship was found in adipose. An overview of these results is displayed in ESM N.

### Fluorophores in excitation-emission matrices

The endogenous fluorophores expected to be observed in samples, as displayed in Table 1, required revision due to discrepancies between expected and observed fluorophores. Consequently, the literature search was extended. Table 4 provides an overview of endogenous fluorophores potentially contributing to the fluorescence peaks observed in EEMs.

**Table 4:** Overview of endogenous fluorophores from literature potentially contributing to observed EEM peaks

Observed ex./em. wl. (nm)	Endogenous fluorophores (ex./em. wl. in nm)	Name assigned
280–300/283.5–383.5	Elastin (285/350 [15]) (290/365 [16])	Peak A
	Tryptophan (295/340–350 [24]) (295, 280/345, 350 [13]) (260–295/325–375 [18])	
	Tyrosine (280/312 [13]) (270/320 [44]) (275/300 [12])	
320–340/335–435	Collagen (340/390 [11]) (345/395 [12]) (337/380–420 [13])	Peak B
	Pyridoxine (332, 340/400 [13]) (310/400 [11]) (310/400 [12])	
355–375/420–520	Kynurenine (380/480 [33]) (365/480 [34]) (360/460–470 [35])	Peak C
	NADH (350/460 13]) (355/475 [11]) (350/470 [12])	
	Protein carbonyls (360/385 [41]) (350/435 [42]) (350/450 [43]) (350/400–500 [23])	
430–450/407–507	Flavins (455/495 [15]) (460/550 [11]) (440–450/540 [14]) (440–450/500–550 [32])	Peak D
450–470/633–733	Unknown	Peak E

ex.: excitation; em.: emission; wl.: wavelength

Peak E was included in fluorophore peak monitoring despite no suitable endogenous fluorophore having been identified via a preliminary literature search, as it was observed in most EEMs and could potentially be an indicator of sample state or degree of decomposition. Lipofuscins/lipofuscin like lipopigments/ceroids, as described by [13], could have been a contributor to peak E, but this could not be substantiated with other scientific literature [10, 12]. Peaks A and C correspond to PROT and FOX peaks respectively. Peak D’s range is based on a single measurement at time point 0 in FFR adipose. Furthermore, six additional peaks observed in one or two EEMs each were excluded from analysis (see ESM O).

#### Decomposition over time by tissue type and treatment

**Fig. 2:**
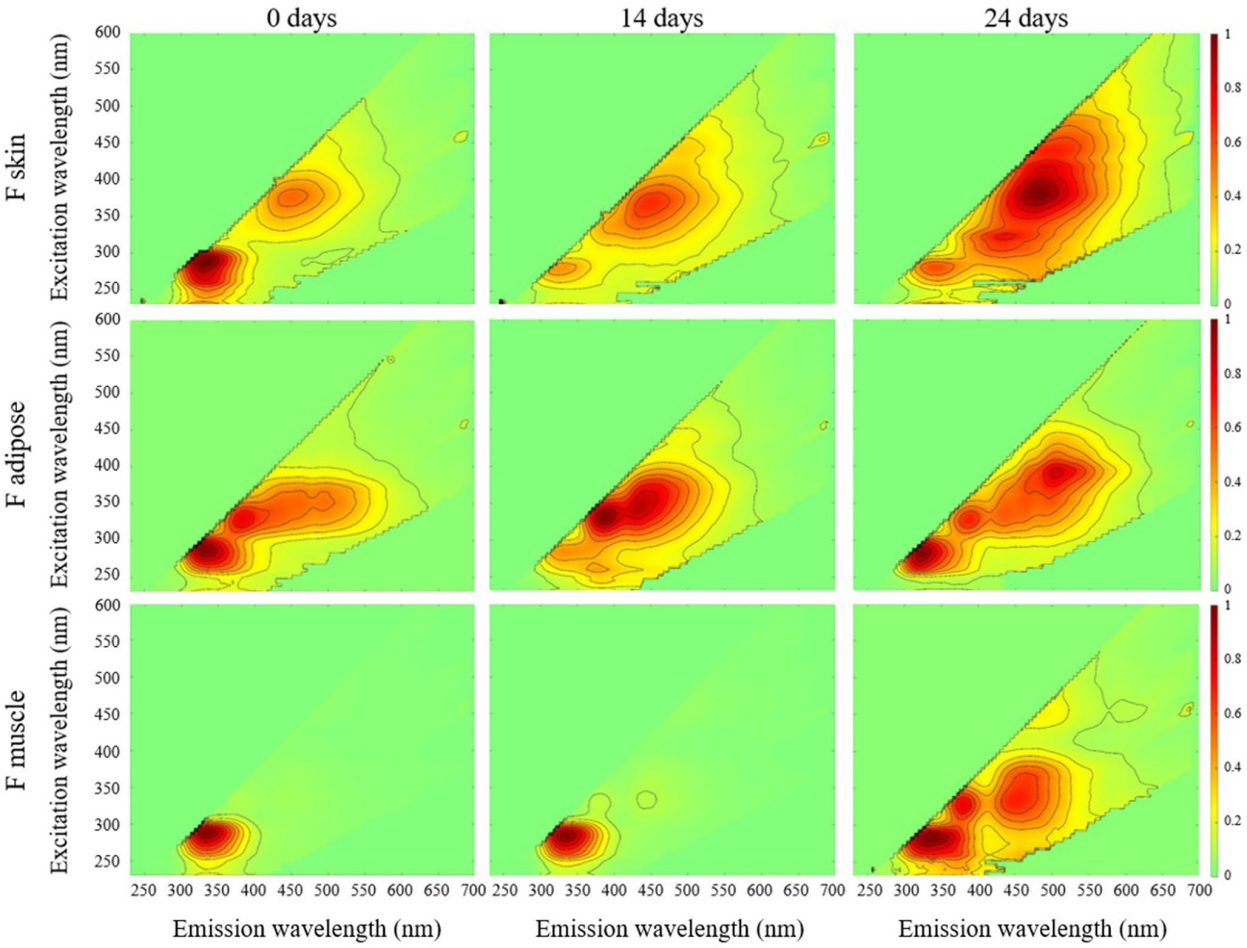
Normalized EEMs for F skin, adipose, and muscle at time points 0, 14, and 24 days (n = 1 for each EEM)

**Fig. 3:**
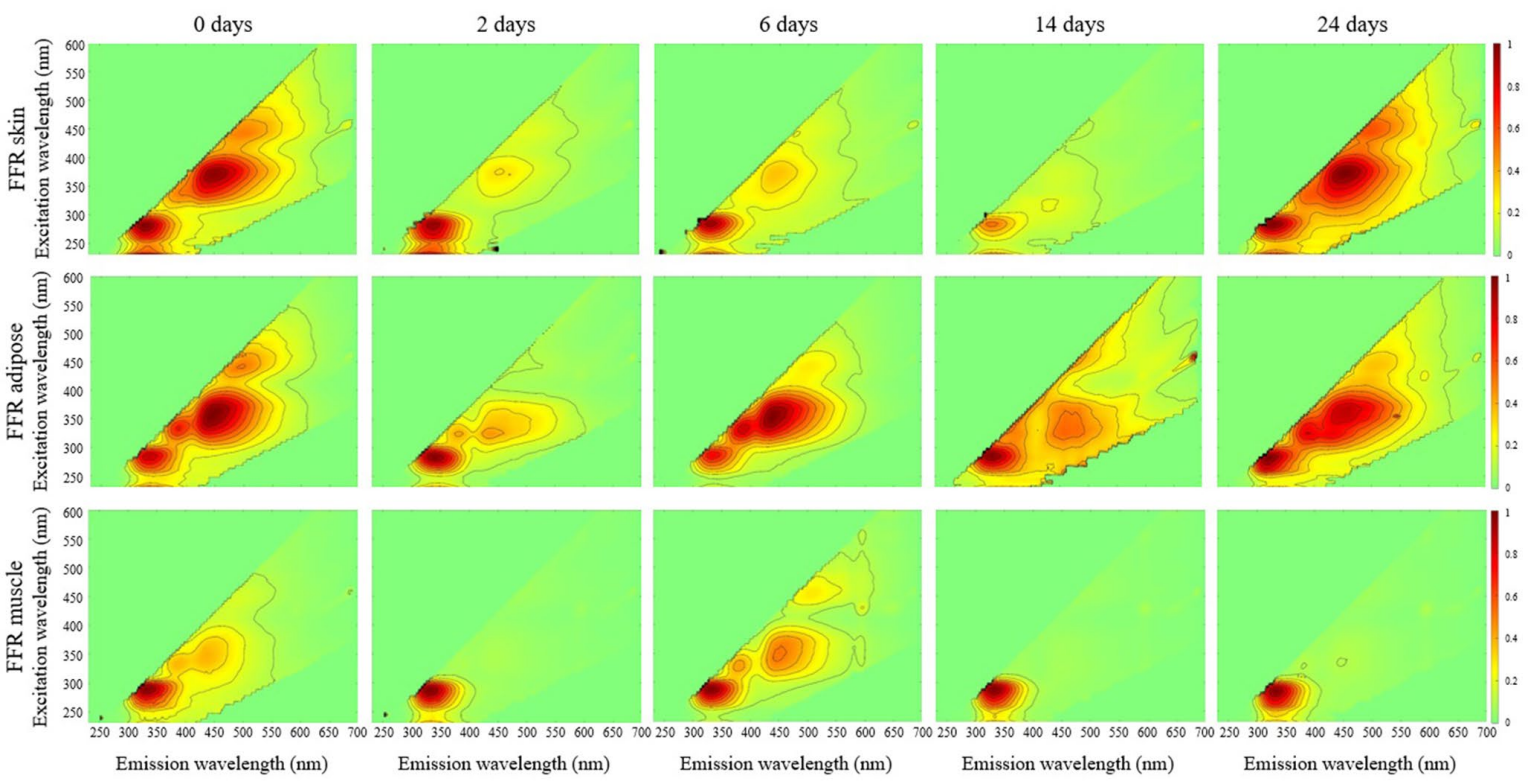
Normalized EEMs for FFR skin, adipose, and muscle at time points 0, 2, 6, 14, and 24 days (n = 1 for each EEM)

Patterns of occurrence in time-dependent peaks, referring to peaks appearing either earlier or later in the decomposition process, were monitored. An overview of fluorophore peak occurrences is shown in ESM P. See Figs. 2 and 3 for the normalized EEMs of F and FFR tissue over time.

In skin, no peak showed time dependence in FFR samples, while two displayed time dependence in F samples. Peak B was only present at time point 24 in F skin, while peak E occurred at time points 0 and 14, with no discernible difference in fluorescence intensity between these time points.

One fluorophore peak displayed time dependent patterns in FFR adipose: peak E. This peak appeared only at time points 14 and 24 in FFR adipose, and showed a ~ 60% decrease in (fluorescence) intensity between these time points. On the other hand, no peak showed time dependence in the F adipose group.

Unlike in adipose, F muscle exhibited more time-dependent peaks than FFR samples. Peak E showed opposing patterns of occurrence, appearing only in undecomposed FFR muscle, and exclusively at time point 24 in F muscle. Peaks B, C, and D also showed time dependence in F muscle, with the same pattern of occurrence as peak E.

#### Fresh vs. fresh-frozen

A fluorophore peak can help distinguish between F and FFR samples if a peak is consistently found across all time points in one group, and is absent from the other.

In skin, peak A was consistently found across all time points in both treatments, while no peak was consistently absent across samples in either treatment group.

In adipose, peaks B and E were the only peaks consistently found in all samples in the F group, while peaks A and B were consistently present in all samples in the FFR group. Peak C was the only peak to be absent across all time points in F adipose. No one peak was present at all time points in one treatment group while being absent from all time points in the other group.

Similarly to skin samples, peak A was consistently present at all time points in both F and FFR muscle. No peak was consistently found in one treatment group while being absent from all samples in the other group.

### Multiple freeze-thaw cycles

The effect of one vs. two F-T cycles on PROT and FOX fluorescence was examined after samples from time point 2 in the FFR group were re-frozen following initial measurements, and later had to be measured again. Graphs illustrating the fluorescence intensity ratio of PROT and FOX before and after a second F-T cycle for each tissue type are displayed in ESM Q.

PROT levels tended to be less impacted by a second F-T cycle compared to FOX. Average levels of PROT and FOX appeared to be less affected by F-T cycles overall in skin samples, with PROT ratios varying from being 20% lower to 3% higher, while FOX fluctuated between being 3% and 27% lower. Additionally, both fluorophores tended to display higher fluorescence intensities following re-freezing.

## Discussion

### Temperature, precipitation, and qualitative observations of decomposition

Although ADD did not match exactly between F and FFR hands at the same time points, F hands at time point 14 had a lower ADD compared to FFR hands at time point 20, while FFR hands at time point 24 had an ADD above 300 ADD, with only F time point 24 displaying a similar ADD. Therefore, meaningful time point comparisons could still be made within the context of this experiment. The impact of varying rates of ADD increase, which differed between treatment groups due to fluctuations in weather conditions, on decomposition is uncertain. On average, the FFR group experienced 12 ADD, while the F group experienced 8.5 ADD. Despite a lack of literature on how the rate of change in ADD affects decomposition, as the average difference was only of 3.5 °C, it may be reasonable to conclude that this difference had a negligible impact on sample decomposition. Burial likely also had some dampening effect on unpredictable weather conditions, as shallow burial temperatures trail surface temperatures by approximately 12 h [47, 48].

Similarly to ADD, while total precipitation did not correspond exactly between F and FFR hands at the same time points, total precipitation for time point 14 F hands was lower than for time point 20 FFR hands, and the total precipitation for time point 24 F and FFR hands differed only by 6.5 mm. Hence, relevant time point comparisons could still be made within the scope of this experiment.

Although the discrepancies in rate of change in ADD and total precipitation likely did not have a significant effect on decomposition, it is preferable to maintain a high level of consistency in these variables between treatment groups, as temperature and moisture are known to tangibly affect the decomposition of remains [3, 49, 50].

At time point 14, FFR hands exhibited brown discoloration at the severance points, potentially indicating earlier desiccation than F hands, which did not display such discoloration nor signs of drying. However, this observation is limited in that it is based on the comparison of four hands from two separate donors, and so could stem from intrinsic differences in the donors themselves rather than treatment effects.

### Fluorescence spectroscopy

#### Decomposition over time by tissue type and treatment

Decomposition trends varied primarily by treatment group, with F samples generally aligning with the expectation that PROT levels would decrease over time while FOX increased, except for skin, which showed a decrease in FOX. By contrast, FFR samples deviated significantly from these expectations and displayed differing trends across tissue types. In all instances, a quadratic trend line provided a better fit for F datasets than FFR. The discrepancy observed in F skin FOX, which decreased over time, could, in part, be a result of the challenges faced when working with dermal samples. This tissue type was directly exposed to soil and any microorganisms therein, hence, fluorescence measurements may include light emitted by these organisms despite cleaning with water. This may be reflected in the higher fluorescence intensities of both PROT and FOX in skin compared to other tissue types. FFR muscle, unlike FFR adipose and skin, defied expectations across all parameters: a significant increase in PROT at time point 24, an initial increase in FOX followed by a decrease, and a slight increase in the PROT-FOX ratio were observed. It may be that as muscle fibers denature over time, a larger surface area of tissue becomes exposed, thereby increasing the fluorescence intensity of PROT over time despite decomposition. However, this mechanism would, theoretically, entail a corresponding increase in FOX over time, which was not observed.

Graphing PROT-FOX data and assessing relationship significance between time points effectively established decomposition trends in F and FFR tissues. Although not all trend lines, particularly for FFR data, fit the data well, this approach provided a more comprehensive overview than using only one of these methods. Increasing the number of time points tested, especially at a larger ADD, and between time points 0 and 14 for F samples, would enhance understanding of decomposition patterns. Exploring clustering on a third variable prior to trend line fitting could improve understanding of decomposition over time for either treatment group. Nevertheless, the data clearly indicated that F tissue decomposition followed initial expectations, while FFR tissue did not, and fluctuated more over time and across tissue types. As differing patterns are present across all three FFR tissue types, this suggests that the discrepancies observed arise from an intrinsic difference in the decomposition of F and FFR tissue. Analyzing trends in PROT and FOX levels over time therefore clearly demonstrated that a difference exists in the taphonomic processes undergone by F and FFR tissue, and that these vary slightly depending on tissue type.

#### Fresh vs. fresh-frozen

Upon evaluation of the significant differences between F and FFR samples at corresponding time points, significant differences were found for at least two parameters for every tissue type, except for the comparison of skin samples at time point 14. The pattern of significance observed suggests that time is an important factor in differentiating between sample states due to fluctuations in decomposition over time. At time point 14, for every tissue type, at least one parameter resulted in no significant difference between treatment groups. This appears to arise from the intrinsic decomposition patterns of F and FFR tissue, whereby time point 14 data points tend to overlap between treatment groups. Just one non-significant relationship between treatment groups was found in adipose, indicating a high potential for this tissue to be used in distinguishing F from FFR samples.

The results of the PROT-FOX measurements, and subsequent ANOVAs, clearly indicated a difference in the taphonomic process undergone by F and FFR remains, as well as different tissue types. Additional time points should be investigated with a larger sample size to substantiate these findings and ascertain whether fluorescence measurements in adipose could distinguish F from FFR material, regardless of the length of time the remains have been buried.

### Fluorophores in excitation-emission matrices

#### Decomposition over time by tissue type and treatment

Patterns of occurrence in fluorophore peaks varied by tissue type and treatment group, with some peaks reflecting time-dependent decomposition patterns. In most instances, at least one fluorophore peak exhibited clear time-dependent patterns for a given tissue type/treatment group. One peak displayed time dependence in both treatment groups for a given tissue type: peak E in muscle samples. The patterns of occurrence for this peak were opposite between treatment groups. Some peaks also displayed time-dependence in one treatment group, but not in the other group for the same tissue type.

As only one EEM per time point was generated per tissue type/treatment group, this limits the conclusions that can be drawn regarding the occurrence of fluorophore peaks, and how these may exhibit different decomposition patterns in F and FFR human tissue. However, preliminary results indicate that the occurrence of fluorophore peaks over time differ based on tissue type and treatment group.

#### Fresh vs. fresh-frozen

The presence or absence of certain fluorophore peaks in an EEM may be useful in discriminating between F and FFR samples in practice if a peak is consistently found across all time points in one treatment group, and is absent from the other group. None of the five peaks examined fulfilled these criteria. In some instances, a certain peak was present or absent across all time points for one treatment group, but the opposite was never the case in the other group. Thus, monitoring of fluorophore peaks in EEMs does not appear to be useful in differentiating F from FFR samples, regardless of tissue type. Nevertheless, the differing trends in peak occurrence between F and FFR samples demonstrated that variations in taphonomic processes arise based on sample state.

### Multiple freeze-thaw cycles

The initial freezing, and later re-freezing, of time point 2 FFR samples provided an opportunity to explore the effects of multiple F-T cycles on PROT and FOX fluorescence. Results revealed varying impacts of multiple F-T cycles on individual fluorophores: PROT fluorescence intensities fluctuated less on average compared to FOX, with results also varying by tissue type. Following re-freezing, both fluorophores displayed higher fluorescence intensities, and varied less on average in skin, and most in muscle samples. These findings align with previous research by [51] who noted a marked increase in protein carbonyl content with each subsequent F-T cycle. Contrary to the results of [25] and [26], PROT fluorescence tended to increase after a second F-T cycle. The cause of this deviation is unclear: it may be a difference inherent to human tissue, originate from an intrinsic characteristic in the FFR time point 2 donor, or stem from the context of this experiment wherein limbs were buried and left to decompose. Nonetheless, these results highlight that multiple F-T cycles have a differential effect on individual fluorophores, and that this effect varies across tissue types.

### Limitations and future research

This study’s limitations primarily stem from challenges inherent to pilot and human taphonomy research, including an absence of validated protocols and difficulties in sample acquisition. It should also be noted that the fluorescence data does not meet the assumption of independence of the ANOVA test, as three different areas were measured for each sample. The within-group significance results presented should therefore be interpreted with caution.

Only a total of 22 hands, just six of which belonged to the F group, were obtained for analysis. Despite this being a small sample size statistically-speaking, obtaining human material for research, especially fresh material, is extremely difficult, notwithstanding any exclusion criteria. Due to these difficulties, FFR hands were buried and exhumed before F hands were buried, leading to unequal rates of ADD increase between treatment groups. Unfortunately, little is known as to how fluctuations in ADD rates affect decomposition, however, as the difference in average daily ADD was small, this discrepancy likely had an indiscernible effect on decomposition. Soil aeration was changed during exhumation and re-burial of F hands, likely altering anaerobic-driven putrefaction, while abrupt temperature changes due to relocation may have induced condensation on hand surfaces, potentially accelerating decomposition [3, 49, 52]. As these changes affected all samples within a treatment group, it is difficult to ascertain if, and to what extent, these affected fluorescence in F hands. Furthermore, scavenging of time point 14 F hands could have influenced their decomposition. Despite a lack of visible physical damage, unearthing exposed the hands directly to air, precipitation, and additional invertebrates/microorganisms, potentially accelerating the oxidation of PROT to FOX. Although fluorescence measurements for F hands fell within the expected range for this experiment, the possibility that re-burial and scavenging impacted their decomposition cannot be excluded. FFR samples spent, on average, more time in the fridge prior to fluorescence measurements compared to F samples. Time was spent refining LS55 settings during this pilot study to avoid measurement saturation, while ensuring a sufficient signal for AUC calculations. As delays between sampling and measuring may occur in any experiment or forensic investigation, further research into post-sampling changes arising from extended storage periods is needed. Additionally, samples were not homogenized before fluorescence measurements, potentially introducing differences based on tissue structure. Despite its limitations, this study clearly demonstrates that differences exist in the decomposition of F and FFR tissue, and that the number of F-T cycles have a differential effect on PROT and FOX fluorescence which vary by tissue type.

Several aspects of this research warrant further investigation. More time points should be studied, especially for F samples, to better understand decomposition in skin, adipose, and muscle, and capture any discrepancies between treatment groups. Increasing sample size per time point and treatment group, with a variety of donors, should also increase statistical power. Rate of ADD increase and precipitation, exhumation and re-burial, and post-sampling conditions and length of storage warrant further exploration to assess their effects on experimental outcomes. Additionally, further exploration of the impact of multiple F-T cycles on fluorescence intensity of PROT and FOX across tissue types would be beneficial when considering casework application. Assessing the utility and necessity of sample homogenization prior to fluorescence measurements in visualizing differences between F and FFR tissue and tissue types may also be beneficial. Investigation of the aforementioned variables would aid in assessing their impact on the methods applied in this experiment, and thereby determine the methods’ suitability for forensic casework. Additionally, varying depths of burial may be considered as the focus of future studies, although the aim to mimic the disposal of human remains by a perpetrator is best fulfilled by modeling shallow burial [53–56]. Although the behavior of fluorophore peaks in EEMs could be further explored, preliminary findings suggest that these have a limited use in distinguishing between F and FFR samples, and establishing decomposition trends in various tissue types. This method should therefore not be prioritized for further investigation. While sourcing samples, especially fresh ones, will continue to pose challenges for future experiments, it is imperative to strive for consistency in all relevant variables across treatment groups. This will ensure that meaningful comparisons and robust conclusions can be drawn from multiple studies.

## Conclusion

Visual observations of decomposition indicated that fresh-frozen hands may begin to undergo desiccation earlier than fresh hands. Decomposition was quantitatively assessed via protein and FOX fluorescence, and excitation-emission matrix measurements.

Fluorophore peak monitoring in excitation-emission matrices did not appear useful in differentiating fresh from fresh-frozen samples, as no peak showed contrasting patterns of occurrence across treatment groups. On the other hand, protein-FOX measurements showed promise in differentiating fresh from fresh-frozen samples with at least one parameter (protein area under the curve, FOX area under the curve, and/or protein-FOX ratio) displaying a significant difference at corresponding time points between treatment groups.

Protein-FOX measurements suggested that decomposition trends varied mainly based on treatment group, whereby fresh samples generally aligned with the expectation that protein levels would decrease over time while FOX increased. By contrast, fresh-frozen samples deviated significantly from these expectations and displayed different trends based on tissue type. Monitoring of time-dependent fluorophore peaks in excitation-emission matrices revealed differing decomposition trends based on both tissue type and treatment group, but did not display a clear difference between treatment groups for a given tissue type.

The limitations encountered in this study stem predominantly from challenges inherent to pilot and human taphonomy studies, whereby validated protocols have yet to be developed, and samples are difficult to source. Issues included difficulty sourcing fresh samples resulting in fresh-frozen hands being buried much earlier compared to fresh hands, leading to varying rates of accumulated degree day increase between treatment groups, and prolonged fridge storage of fresh-frozen hands prior to fluorescence measurements. Nonetheless, this study demonstrates that differences in fresh and fresh-frozen human tissue decomposition exist, and that these vary by tissue type. This has great repercussions on future taphonomic research, as frozen material can no longer be assumed to accurately represent decomposition in fresh tissue for all measurement types.

## Electronic supplementary material

Below is the link to the electronic supplementary material.

Supplementary Material 1

## Data Availability

Data are available upon reasonable request to the authors.
