# Supplementary material for: Fluorescence of various buried fresh and fresh-frozen-thawed tissue types up until the point of active decay: a human taphonomy study
Source: Int J Legal Med. 2024 Dec 20;139(2):917–30. doi: 10.1007/s00414-024-03387-w (PMC11850421; doi:10.1007/s00414-024-03387-w)
Supplement: Supplementary file 1 — Supplementary Material 1 [file 414_2024_3387_MOESM1_ESM.docx]

**Electronic Supplementary Material**

**Fluorescence of various buried fresh and fresh-frozen-thawed tissue types up until the point of active decay: A human taphonomy study**

*International Journal of Legal Medicine*

Authors: Emmanuelle Charlot ^1^, Anas Gasser ^2^, Roelof-Jan Oostra ^1^, Maurice C. G. Aalders ^2, 3^, Tristan Krap ^1, 2, 4^

Corresponding author: T. Krap, t.krap@maastrichtuniversity.nl

E. Charlot: https://orcid.org/0009-0005-1359-7998
A. Gasser: <https://orcid.org/0000-0002-0688-4420>
R-J. Oostra: https://orcid.org/0000-0002-2452-8307
M. Aalders: <https://orcid.org/0000-0001-8083-9209>
T. Krap: <https://orcid.org/0000-0003-2438-3694>

Affiliations:

^1^ Department of Medical Biology, Section Clinical Anatomy and Embryology, AmsterdamUMC, Location Academic Medical Centre, Meibergdreef 9, 1105 AZ Amsterdam, The Netherlands

^2^ Department of Biomedical Engineering and Physics, Amsterdam UMC, Location Location Academic Medical Centre, Meibergdreef 9, PO Box 22700, 1100 DE Amsterdam, The Netherlands

^3^ Co van Ledden Hulsebosch Center, Science Park – Building 904
(Room C2.243), 1098 XH Amsterdam Amsterdam, The Netherlands

^4^ Faculty of Law and Criminology, Maastricht University, Minderbroedersberg 4-6, 6211 LK, Maastricht, The Netherlands

**A: Donor** **characteristics**

Table S1. *Donor sex and age in years at time of death*

| **Donor number (arbitrary)** | **Sex** | **Age at time of death (years)** |
| --- | --- | --- |
| 1 | M | 97 |
| 2 | F | 86 |
| 3 | M | 72 |
| 4 | F | 85 |
| 5 | F | 67 |
| 6 | M | 79 |
| 7 | M | 65 |
| 8 | M | 66 |
| 9 | F | 77 |
| 10 | M | 78 |
| 11 | M | 66 |

M: male; F: female


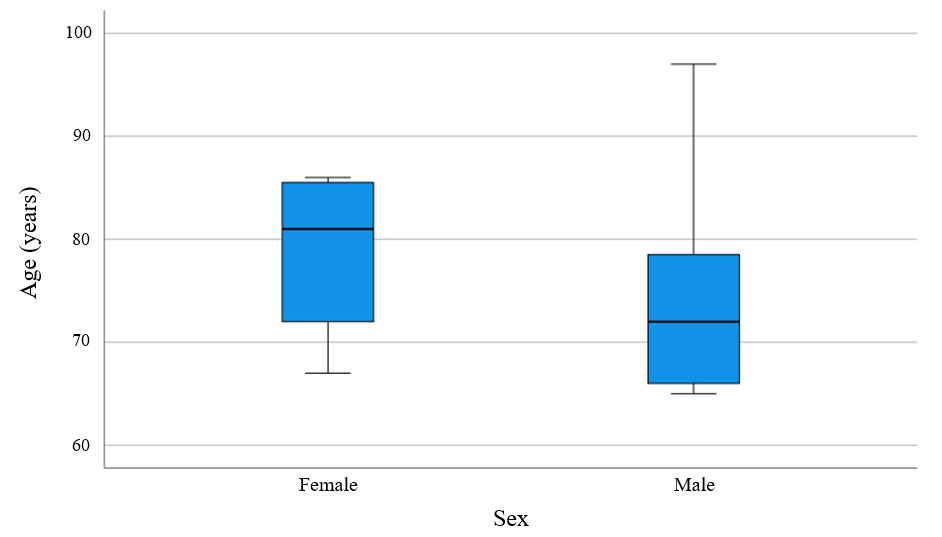


*Figure S1.* Boxplot of donor age (in years) grouped by sex

**B: Supplementary water provided to burial pits**

Table S2. *Supplementary water provided to burial pits*

| **Date of watering (dd/mm/yyyy)** | **Rainfall (mm)*** | **Area watered (m²)** | **Amount of water (L)** | **Pits watered (treatment group/time point in days)** |
| --- | --- | --- | --- | --- |
| 20/09/2022 | 2.1 | 0.49 | 5.5 | FFR/all |
| 22/09/2022 | 0 | 1 | 6 | FFR/4, 6, 9, 14, 20, 24 |
| 04/10/2022 | 0 | 1 | 6 | FFR/20, 24 |
| 07/10/2022 | 0 | 1 | 7 | FFR/20, 24 |
| 12/10/2022 | 0 | 1 | 7 | FFR/24 |
| 03/11/2022 | 3.4 | 1 | 6 | F/24 |
| 11/11/2022 | 0 | 1 | 7 | F/all |
| 14/11/2022 | 0 | 1 | 7 | F/all |
| 05/12/2022 | 0.7 | 0.47** | 1.5 | F/all |
| 09/12/2022 | 0.1 | 0.23** | 0.6 | F/24 |

* Rainfall measurements sourced from [1]

** Areas based on the dimensions of the plastic bins which contained the F hands following relocation.

**C: Exhumation scheme**

Table S3. *Date of interment and exhumation for each hand pair*

| **Date interred (dd/mm/yyyy)** | **Date exhumed (dd/mm/yyyy)** | **Time point (days)** | **ADD** | **Treatment group** |
| --- | --- | --- | --- | --- |
| 20/09/2022 | 22/09/2022 | 2 | 31.7 | FFR |
| 20/09/2022 | 24/09/2022 | 4 | 58.7 | FFR |
| 20/09/2022 | 26/09/2022 | 6 | 82.8 | FFR |
| 20/09/2022 | 29/09/2022 | 9 | 115.6 | FFR |
| 20/09/2022 | 04/10/2022 | 14 | 179.9 | FFR |
| 20/09/2022 | 10/10/2022 | 20 | 257.6 | FFR |
| 20/09/2022 | 14/10/2022 | 24 | 300.4 | FFR |
| 03/11/2022 | 12/12/2022 | 24 | 340.4 | F |
| 11/11/2022 | 09/12/2022 | 14 | 196.9 | F |

**D: Hand sampling diagram**

Blank diagrams obtained from Lameira et al. ^[2]^.


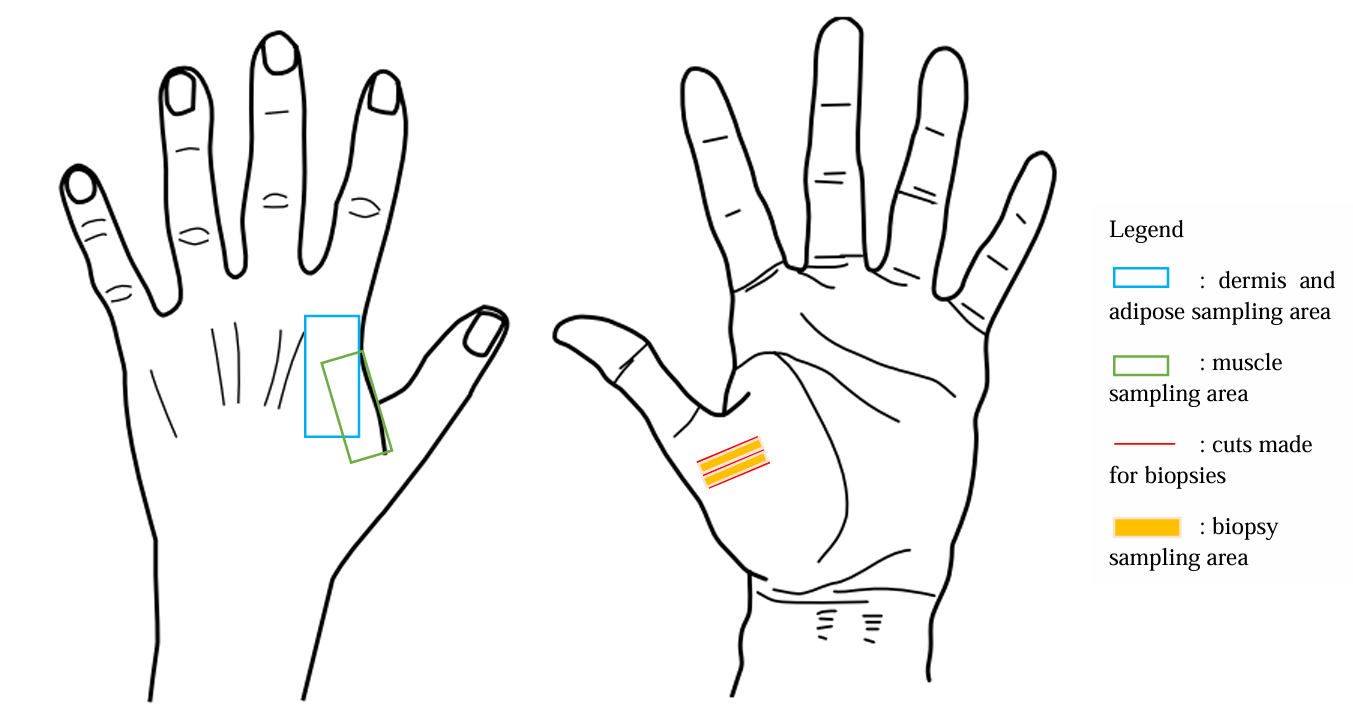


*Figure S2.* Diagram of tissue sampling areas on hands

**E: LS55 fluorescence spectrometer measurement settings**

Table S4. *LS55 settings for fluorescence measurements by tissue type*

| **Tissue type** | **Excitation slit (nm)** | **Emission slit (nm)** | **Gain** |
| --- | --- | --- | --- |
| Skin | 15 | 20 | Medium |
| Adipose | 15 | 12 | Medium |
| Muscle | 15 | 12 | Medium |

Excitation wavelengths were chosen based on the recommendations of [3].

Table S5. *LS55 settings for PROT and FOX fluorescence measurements*

| **Measurement type** | **Excitation wavelength (nm)** | **Emission range (nm)** | **Step size (nm)** | **Scanning speed (nm/min)** |
| --- | --- | --- | --- | --- |
| PROT | 285 | 300 – 500 | 0.5 | 300 |
| FOX | 370 | 400 – 600 | 0.5 | 300 |

EEM measurements utilized the same slit sizes and gain as individual PROT-FOX measurements, with a scanning speed of 1 500 nm/min, and a range of 230 nm to 600 nm for excitation wavelengths, and 230 nm to 700 nm for emission wavelengths. Step sizes of 5 nm and 0.5 nm were used for excitation and emission wavelengths respectively.

**F: Procedure for fluorescence measurements and data analysis using an ND filter**

*Fluorescence measurements*

In case of oversaturation of peaks, a neutral density (ND) filter with optical density (OD) 0.1 was used to evenly reduce the amount of light picked up by the LS55, and provide non-saturated readings using the same settings. In these instances, a white diffuse reflectance standard tile was used to establish a spectrum of transmission for the ND filter. This was achieved by placing the white tile under the optic fiber of the LS55, and measuring its reflection at an excitation wavelength of 330 nm, with an emission range of 300 nm to 400 nm, a step size of 0.5 nm, and scanning speed of 300 nm/min. The slit sizes used were 2.5 nm for both excitation and emission slits. Care was taken to ensure that the signal was not saturated. This process was repeated after placing the ND filter in front of the detector, and taking another measurement. The ND filter was then left in place, and the white tile removed and replaced by a sample of interest, with the optical fiber 0.5 mm above the sample. Fluorescence was then measured using the settings established for PROT or FOX measurements as needed depending on which measurement was saturated. The complement PROT or FOX measurement was then taken after removal of the ND filter.

*Data analysis*

For a given area on a sample where an ND filter had been used, the spectra obtained with the reflectance tile plus the ND filter, and the reflectance tile alone, were entered into MATLAB® and divided by each other to produce a transmission spectrum for the ND filter. This spectrum was then used to scale fluorescence measurements prior to calculation of the various AUCs and PROT-FOX ratio.

**G: Emission wavelength thresholds for calculating PROT and FOX AUCs**

To set thresholds for PROT and FOX AUC calculations, measurements were plotted in Microsoft Excel, and the wavelength range which captured the emission maxima and adjacent regions for the largest number of measurements for time points 0 and 24 was determined, within a given tissue type and treatment group. These were then used to obtain the threshold which would capture the emission maxima and adjacent regions for the majority of measurements for that tissue type.

In the example below, the threshold for PROT measurements in adipose was set by identifying the range that captured the emission maxima and adjacent regions for time points 0 and 24, for F and FFR adipose, individually. The overall lowest and highest limits, 309.5 nm in FFR time point 24, and 349 nm in F time point 0 respectively, were then used as the thresholds for PROT adipose measurements, as this range ensured that the emission maxima and immediate regions of most measurements would be accounted for.

**
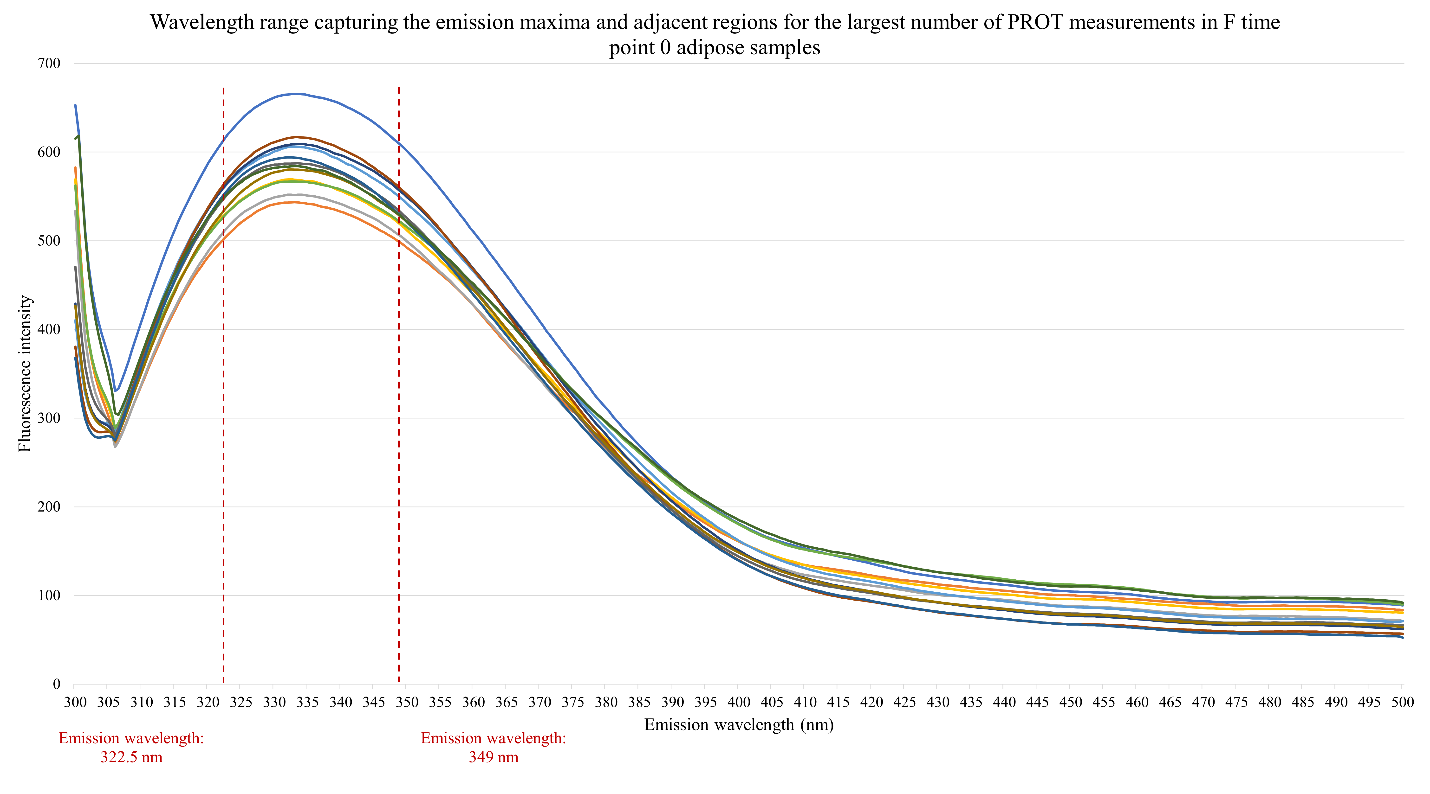
**

*Figure S3.* Line graph displaying the wavelength range capturing the emission maxima and adjacent regions for the largest number of PROT measurements in F time point 0 adipose samples

**
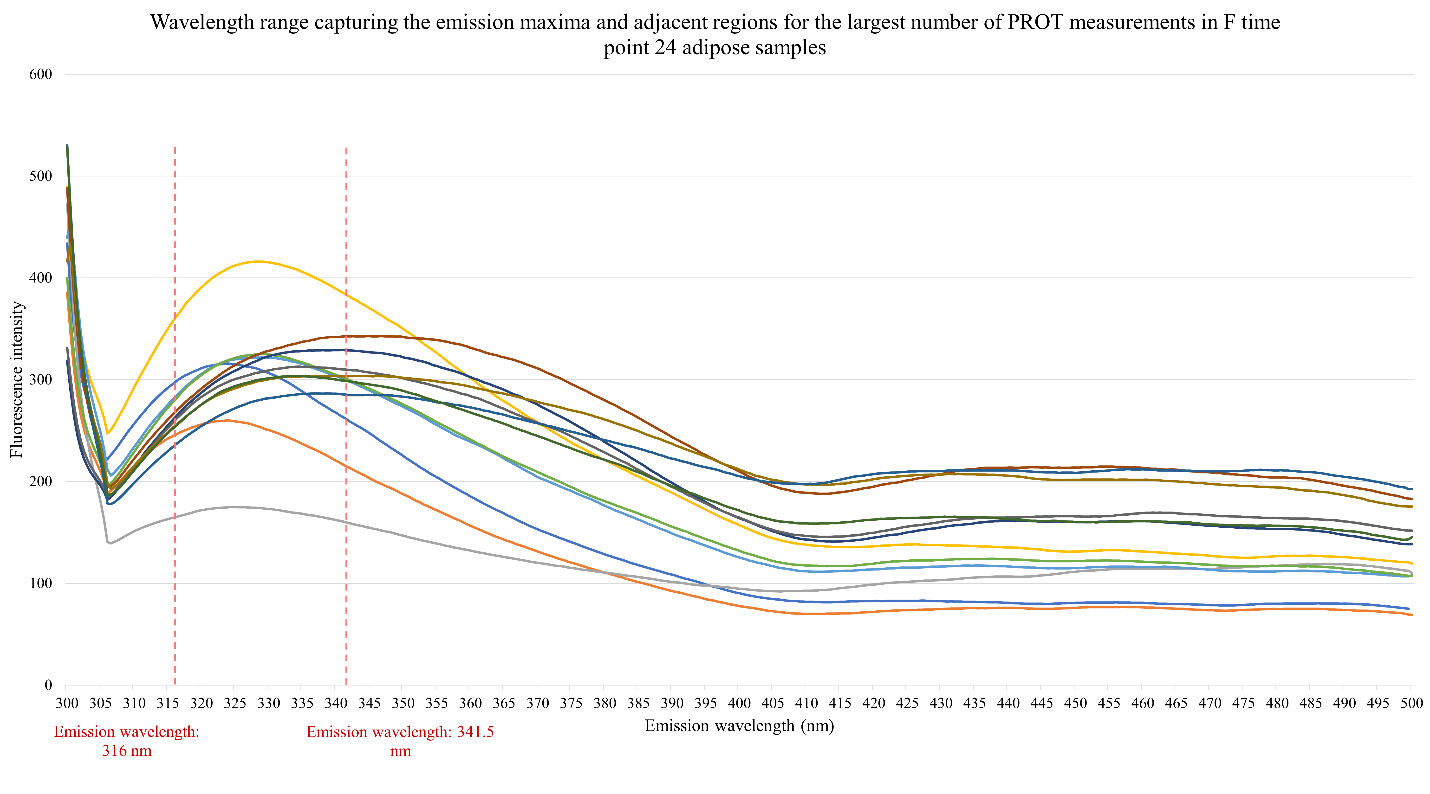
**

*Figure S4.* Line graph displaying the wavelength range capturing the emission maxima and adjacent regions for the largest number of PROT measurements in F time point 24 adipose samples

**
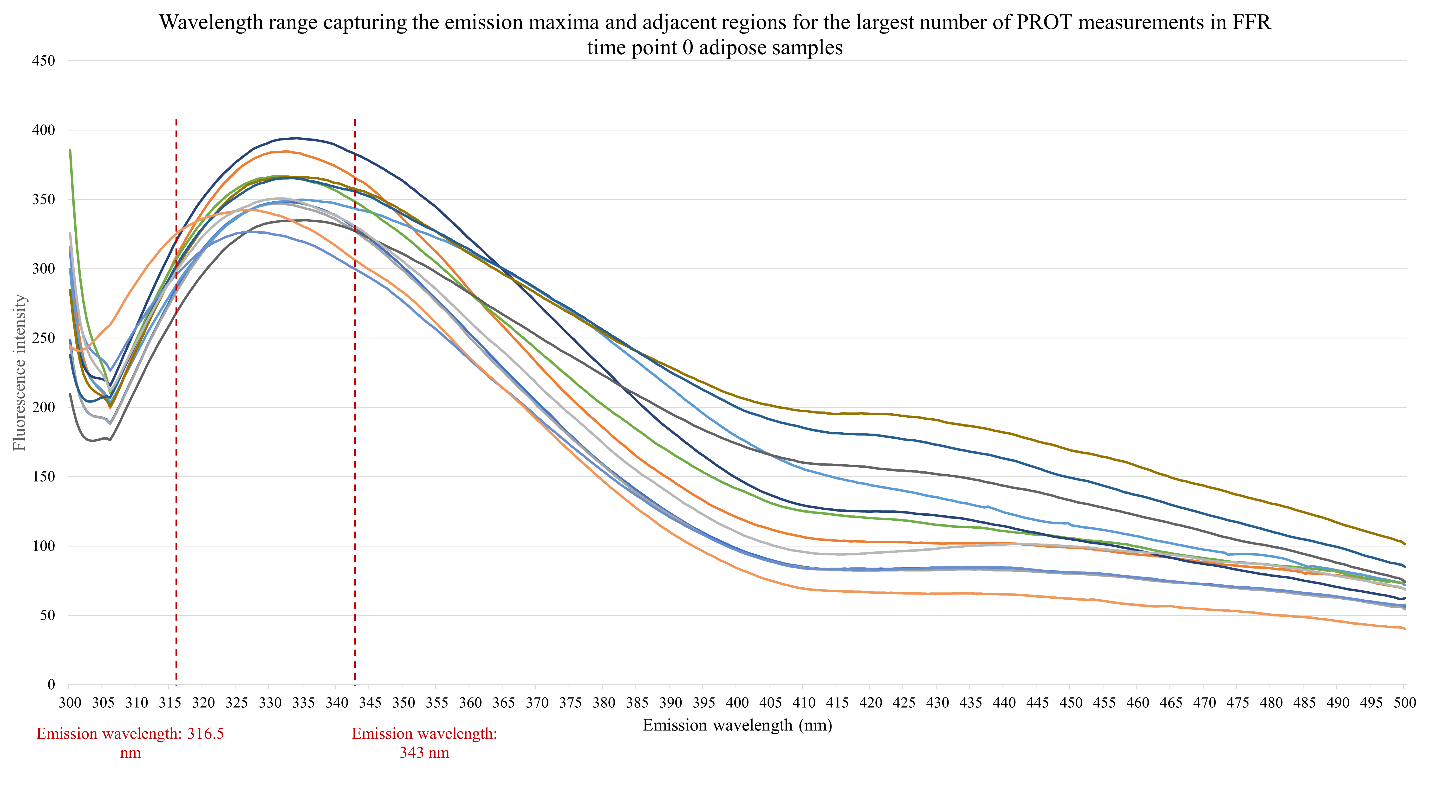
**

*Figure S5.* Line graph displaying the wavelength range capturing the emission maxima and adjacent regions for the largest number of PROT measurements in FFR time point 0 adipose samples

**
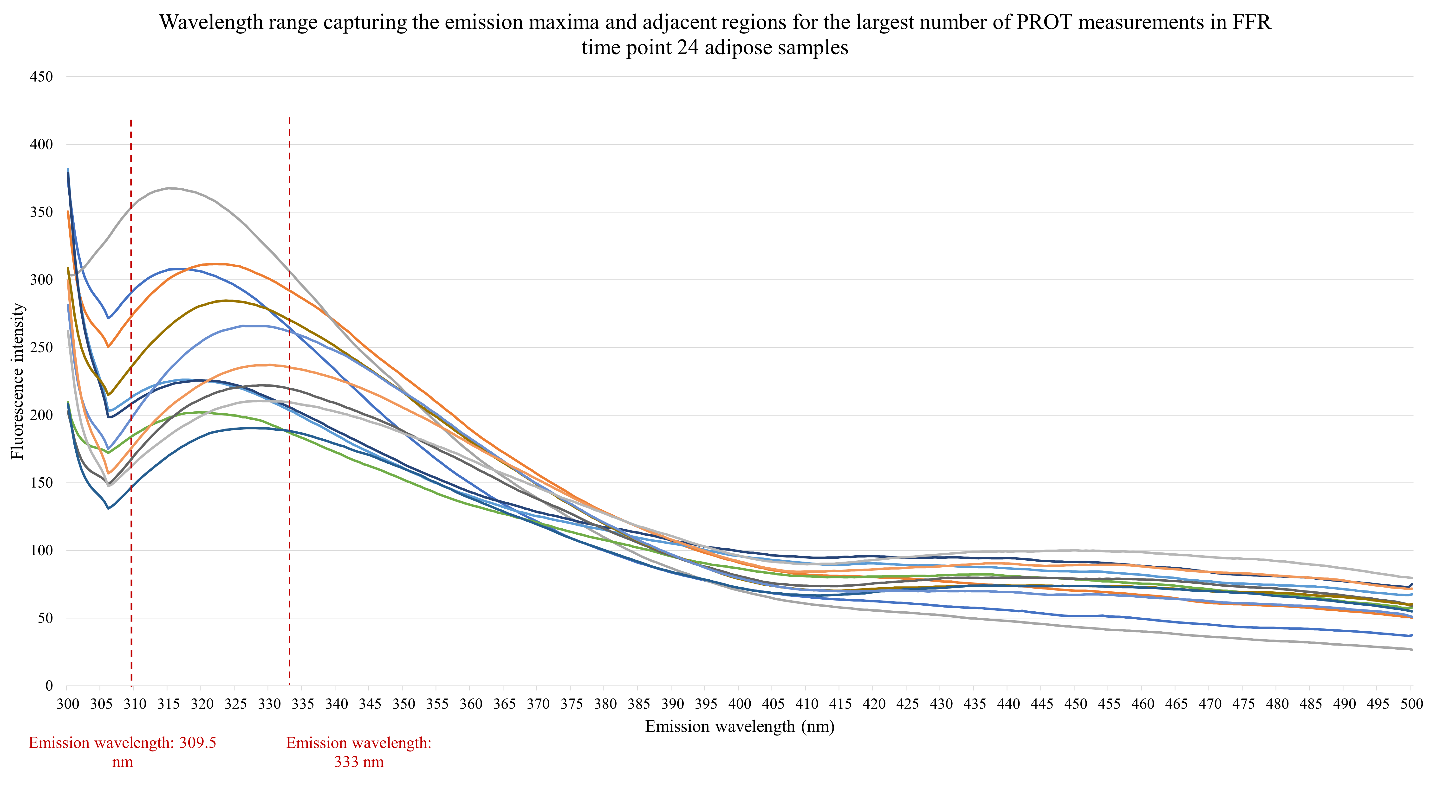
**

*Figure S6.* Line graph displaying the wavelength range capturing the emission maxima and adjacent regions for the largest number of PROT measurements in FFR time point 24 adipose samples

Table S6. *Thresholds for PROT and FOX measurements by tissue type*

| **Tissue type** | **Measurement type** | **Emission wavelength thresholds (nm)** |
| --- | --- | --- |
| Skin | PROT | 313.5 – 353.5 |
| Skin | FOX | 427.5 – 498.5 |
| Adipose | PROT | 309.5 – 349.0 |
| Adipose | FOX | 430.0 – 472.5 |
| Muscle | PROT | 313.5 – 348.0 |
| Muscle | FOX | 430.5 – 484.5 |


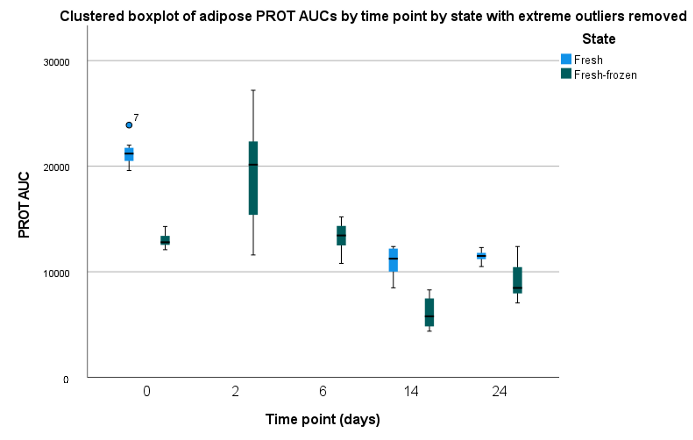
*
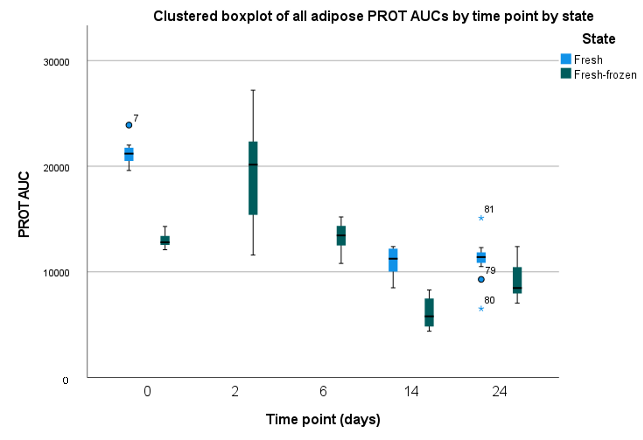
***H: Example of statistical analysis carried out in SPSS®**

(B)

(A)


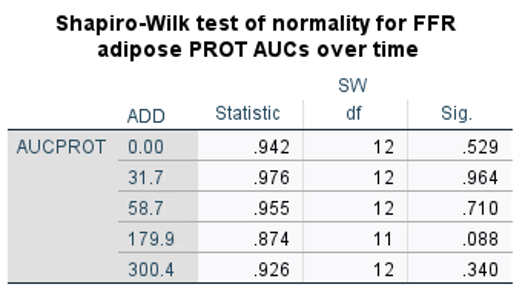

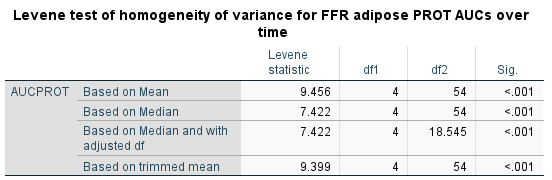


(D)

(C)

(F)


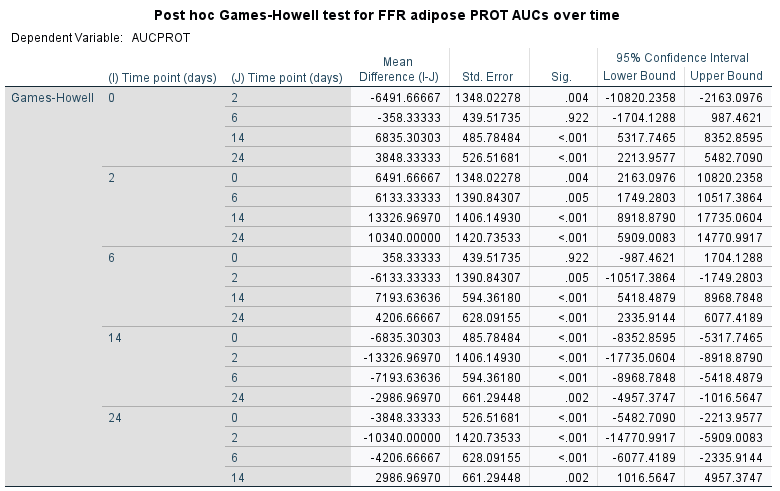

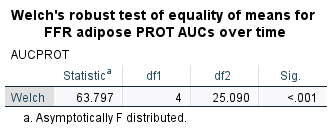


(E)

*Figure S7.* Procedure for testing relationship significance of PROT AUCs over time in FFR adipose in SPSS®: (A) Clustered boxplot of FFR adipose PROT AUCs by time point and treatment (extreme outliers depicted as stars, outliers shown as circles). (B) Clustered boxplot of FFR adipose PROT AUCs by time point and treatment with extreme outliers removed. (C) Shapiro-Wilk test of normality for FFR adipose PROT AUCs over time. P-values (column circled in red) are used to determine normality (p > 0.05 data is normal, p < 0.05 data deviates from a normal distribution). (D) Levene test of homogeneity of variance for FFR adipose PROT AUCs over time. P-value (circled in red) is used to determine homogeneity of variance (p > 0.05 assumption for homogeneity of variance is met, p < 0.05 assumption for homogeneity of variance is not met). (E) Welch’s ANOVA statistic for FFR adipose PROT AUCs over time. P-value (circled in red) is used to determine statistical significance (p < 0.05 a significant difference between group means exists, p > 0.05 a significant difference between group means does not exist). (F) *Post hoc* Games-Howell test for FFR adipose PROT AUCs over time. P-values (column circled in red) are used to evaluate the significance of relationships between multiple groups tested using the Welch’s ANOVA (p < 0.05 a significant difference exists between group means, p > 0.05 there is no significant difference between group means).

**I: Method for identifying fluorophores in EEMs**

To identify fluorophore peaks in EEMs, ranges had to be defined to assess which peaks were present or absent. Fluorescence ranges were derived from the excitation/emission wavelengths observed for the central coordinates of each independent contour of highest fluorescence for a given region at time point 0 of EEMs, for each tissue type and state (see Figs. S8-S9 below for an illustration of this method). The only exception is the FOX peak, where time point 24 EEMs were used to define its range. The median value for the excitation and emission wavelengths respectively for each peak were used to define the ranges. For excitation wavelengths, the range extended ± 10 nm around the median, while for emission wavelengths, it extended ± 50 nm from the median. When needed, the excitation wavelength’s median was rounded to the nearest multiple of 5, reflecting the measurement’s 5 nm step size, or rounded up to the nearest 0.5 for the emission wavelength, corresponding to the measurement’s 0.5 nm step size.

Endogenous fluorophores with optimal wavelengths within these ranges were assigned as potential contributors to each observed peak, and arbitrary names were assigned to these peaks as multiple fluorophores could be contributing to the fluorescence of one peak.

Peak presence/absence in each EEM was determined based on whether coordinates within the independent contour of a peak fell within one of the ranges defined.


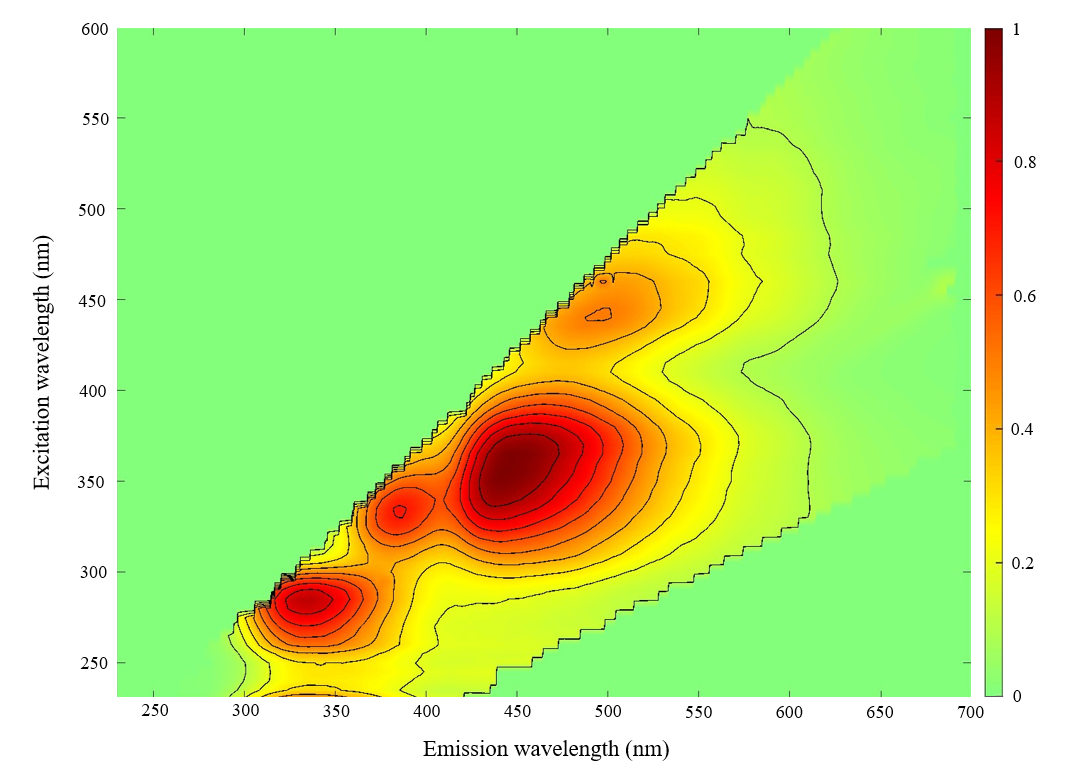


*Figure S8.* Unlabeled normalized EEM for FFR adipose at time point 0


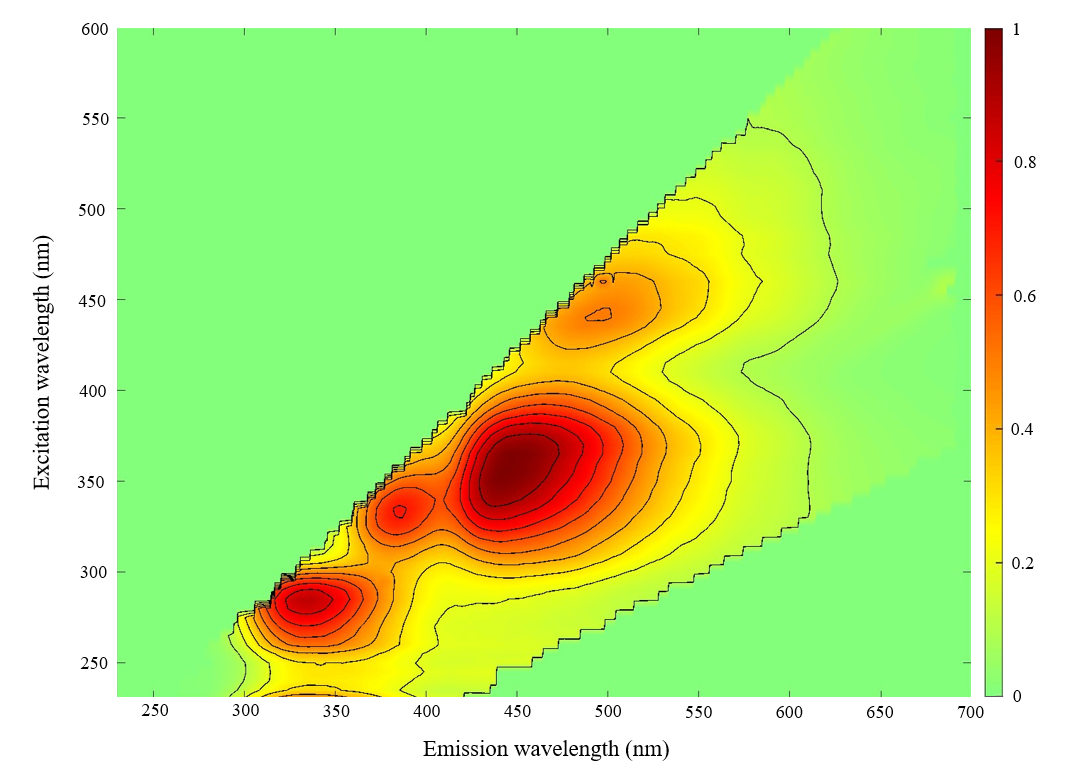


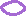

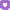

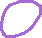

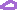


*Figure S9.* Central coordinates (blue dot) recorded for each independent contour of highest fluorescence (purple outline) for a given region in FFR adipose time point 0 normalized EEM

## **J: Precipitation**

Table S7. *Total and average daily* *precipitation (mm) including watering for each FFR and F hand pair*

|  | **FFR hand pairs** | | | | | | | **F hand pairs** | |
| --- | --- | --- | --- | --- | --- | --- | --- | --- | --- |
| Time point (days) | 2 | 4 | 6 | 9 | 14 | 20 | 24 | 14 | 24 |
| ADD | 31.7 | 58.7 | 82.8 | 115.6 | 179.9 | 257.6 | 300.4 | 196.9 | 340.4 |
| Total precipitation (mm) | 5.9 | 43.4 | 47.3 | 124.5 | 169.5 | 195.8 | 236.0 | 142.8 | 242.5 |
| Average daily precipitation (mm) | 2.9 | 10.8 | 7.9 | 13.8 | 12.1 | 9.8 | 9.8 | 4.9 | 6.2 |

## **K: Visual observations of decomposition**

Table S8. *Observations of decomposition for each hand pair by time point and treatment group*

| **Time point (days)** | **ADD** | **Treatment group** | **Observations** |
| --- | --- | --- | --- |
| 0 | 0 | F | Fresh, no signs of decomposition. |
| 0 | 0 | FFR | Fresh, no signs of decomposition. |
| 2 | 31.7 | FFR | Pale color, no skin slippage, *livor mortis* under fingernails, fungal growth mainly on dorsal side of the left hand with some on the fingers of both hands, bloating of left thumb. |
| 4 | 58.7 | FFR | Grey color, no skin slippage, some fungal growth near the wrists. |
| 6 | 82.8 | FFR | White/grey color, some *livor mortis* under the fingers of the left hand, skin is a bit loose but no slippage. |
| 9 | 115.6 | FFR | Pink/white color, skin slippage on dorsal sides and some on the palmar sides of the wrists, patches of dry skin on dorsal sides, brown discoloration at the points of severance which is more extensive on the left hand. |
| 14 | 179.9 | FFR | Green and grey color, extensive skin slippage on dorsal side, some brown discoloration at the points of severance, bloating in both hands. |
| 14 | 196.9 | F | Grey and pink color, no discoloration of extremities, extensive skin slippage. |
| 20 | 257.6 | FFR | White/green color, extensive skin slippage, some drying/browning at the points of severance. |
| 24 | 300.4 | FFR | Extensive skin slippage, some brown discoloration of fingertips and at points severance. |
| 24 | 340.4 | F | Brown discoloration at the points of severance, some dry patches of tissue mainly on the dorsal sides, one loosely attached fingernail remaining on the right hand, all fingernails loosely attached to left hand, thumbs slightly loose due to tissue mass loss, some blue tissue visible on the dorsal side of the left hand (likely a fungus). |

**L: Extreme outliers removed prior to statistical analysis**

Table S9. *Extreme outliers (rounded to three significant figures) removed from datasets prior to statistical analysis*

| **Tissue type** | **Sample name** | **Treatment group** | **ADD** | **Time point (days)** | **PROT AUC** | **FOX AUC** | **PROT-FOX ratio** |
| --- | --- | --- | --- | --- | --- | --- | --- |
| Skin | RD1 | FFR | 31.7 | 2 | 33 700 | 12 900 | 2.60 |
| Skin | RD2 | FFR | 31.7 | 2 | 19 600 | 17 300 | 1.14 |
| Adipose | LA1 | F | 340.4 | 24 | 6 520 | 8 490 | 0.768 |
| Adipose | LA1 | F | 340.4 | 24 | 9 290 | 8 920 | 1.04 |
| Adipose | LA2 | F | 340.4 | 24 | 15 100 | 16 600 | 0.912 |
| Muscle | LM1 | FFR | 300.4 | 24 | 19 600 | 6 000 | 3.26 |

**M:** **Decomposition over time by tissue type by state**

### *Skin*

Table S10. *Relationship significance between time points for FFR skin PROT AUCs*

|  |  |  | ADD | | | | |
| --- | --- | --- | --- | --- | --- | --- | --- |
|  |  |  | 0 | 31.7 | 82.8 | 179.9 | 300.4 |
|  |  | Time point (days) | 0 | 2 | 6 | 14 | 24 |
| ADD | 0 | 0 |  |  |  |  |  |
|  | 31.7 | 2 | 🗸 |  |  |  |  |
|  | 82.8 | 6 | 🗸 | 🗴 |  |  |  |
|  | 179.9 | 14 | 🗴 | 🗸 | 🗸 |  |  |
|  | 300.4 | 24 | 🗸 | 🗸 | 🗴 | 🗸 |  |

🗸: significant (p < 0.05) relationship present; 🗴: significant (p < 0.05) relationship absent

Table S11. *Relationship significance between time points for F skin PROT AUCs*

|  |  |  | ADD | | |
| --- | --- | --- | --- | --- | --- |
|  |  |  | 0 | 196.9 | 340.4 |
|  |  | Time point (days) | 0 | 14 | 24 |
| ADD | 0 | 0 |  |  |  |
|  | 196.9 | 14 | 🗸 |  |  |
|  | 340.4 | 24 | 🗸 | 🗸 |  |

🗸: significant (p < 0.05) relationship present; 🗴: significant (p < 0.05) relationship absent


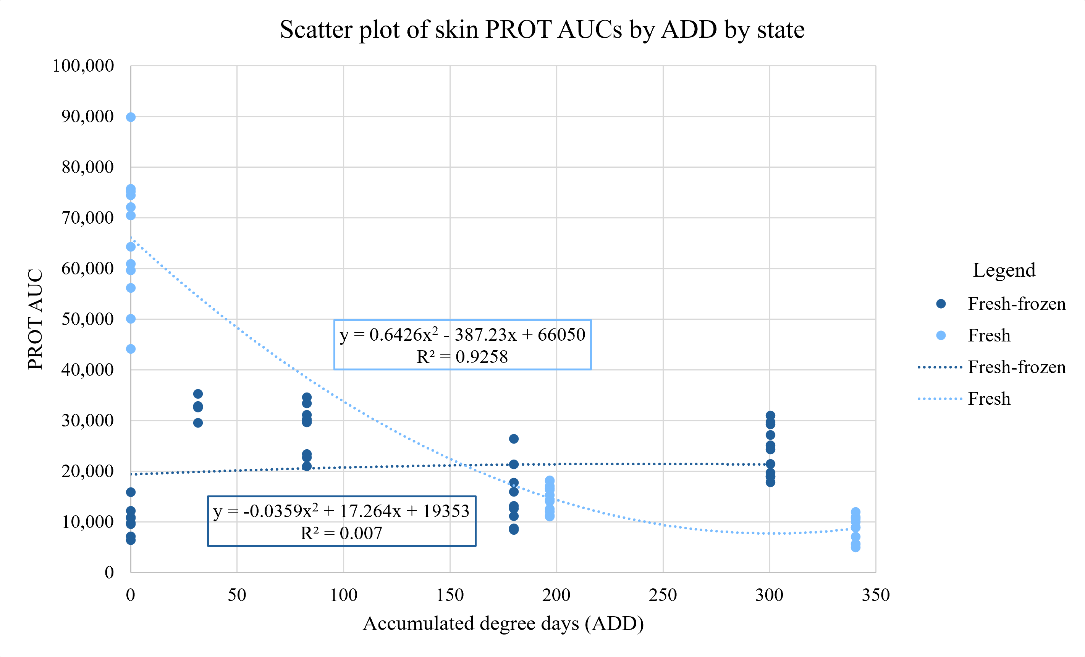


*Figure S10.* Scatter plot of skin PROT AUCs by ADD by state

Table S12. *Relationship significance between time points for FFR skin FOX AUCs*

|  |  |  | ADD | | | | |
| --- | --- | --- | --- | --- | --- | --- | --- |
|  |  |  | 0 | 31.7 | 82.8 | 179.9 | 300.4 |
|  |  | Time point (days) | 0 | 2 | 6 | 14 | 24 |
| ADD | 0 | 0 |  |  |  |  |  |
|  | 31.7 | 2 | 🗴 |  |  |  |  |
|  | 82.8 | 6 | 🗴 | 🗸 |  |  |  |
|  | 179.9 | 14 | 🗴 | 🗴 | 🗴 |  |  |
|  | 300.4 | 24 | 🗴 | 🗸 | 🗸 | 🗸 |  |

🗸: significant (p < 0.05) relationship present; 🗴: significant (p < 0.05) relationship absent

Table S13. *Relationship significance between time points for F skin FOX AUCs*

|  |  |  | ADD | | |
| --- | --- | --- | --- | --- | --- |
|  |  |  | 0 | 196.9 | 340.4 |
|  |  | Time point (days) | 0 | 14 | 24 |
| ADD | 0 | 0 |  |  |  |
|  | 196.9 | 14 | 🗸 |  |  |
|  | 340.4 | 24 | 🗸 | 🗴 |  |

🗸: significant (p < 0.05) relationship present; 🗴: significant (p < 0.05) relationship absent


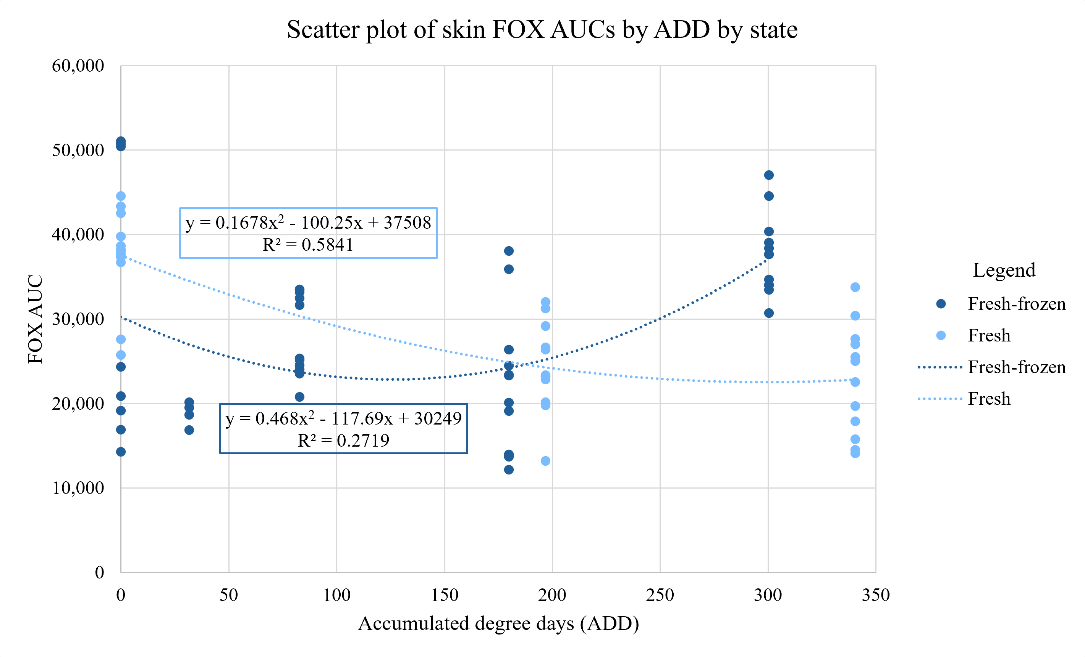


*Figure S11.* Scatter plot of skin FOX AUCs by ADD by state

Table S14. *Relationship significance between time points for FFR skin PROT-FOX ratios*

|  |  |  | ADD | | | | |
| --- | --- | --- | --- | --- | --- | --- | --- |
|  |  |  | 0 | 31.7 | 82.8 | 179.9 | 300.4 |
|  |  | Time point (days) | 0 | 2 | 6 | 14 | 24 |
| ADD | 0 | 0 |  |  |  |  |  |
|  | 31.7 | 2 | 🗸 |  |  |  |  |
|  | 82.8 | 6 | 🗸 | 🗸 |  |  |  |
|  | 179.9 | 14 | 🗴 | 🗸 | 🗸 |  |  |
|  | 300.4 | 24 | 🗴 | 🗸 | 🗸 | 🗴 |  |

🗸: significant (p < 0.05) relationship present; 🗴: significant (p < 0.05) relationship absent

Table S15. *Relationship significance between time points for F skin PROT-FOX ratios*

|  |  |  | ADD | | |
| --- | --- | --- | --- | --- | --- |
|  |  |  | 0 | 196.9 | 340.4 |
|  |  | Time point (days) | 0 | 14 | 24 |
| ADD | 0 | 0 |  |  |  |
|  | 196.9 | 14 | 🗸 |  |  |
|  | 340.4 | 24 | 🗸 | 🗸 |  |

🗸: significant (p < 0.05) relationship present; 🗴: significant (p < 0.05) relationship absent


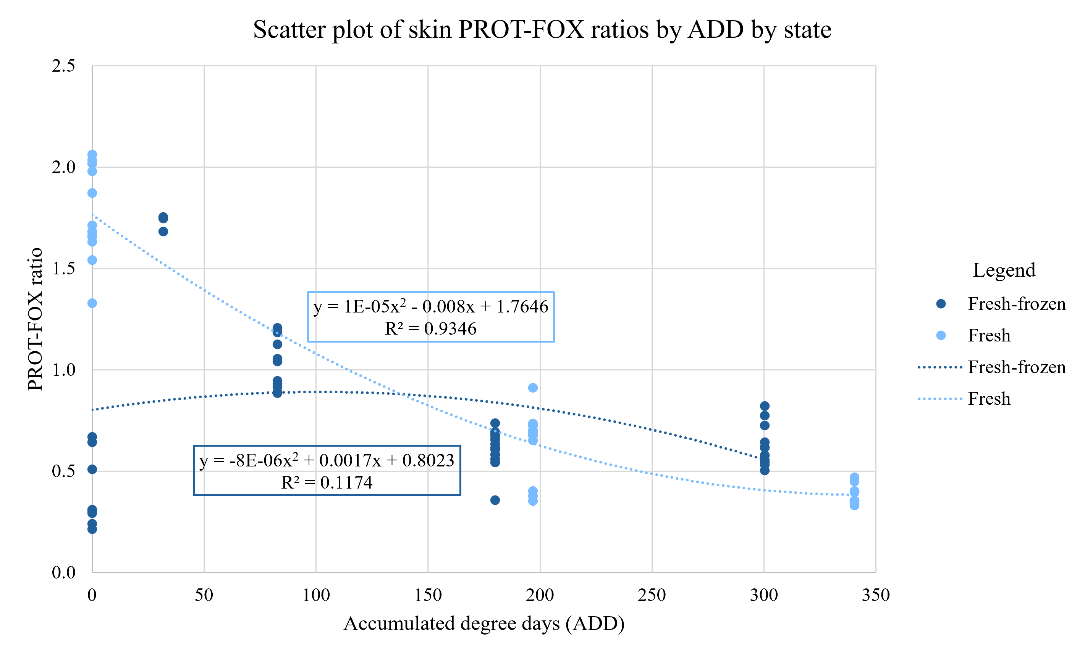


*Figure S12.* Scatter plot of skin PROT-FOX ratios by ADD by state

*Adipose*

Table S16. *Relationship significance between time points for FFR adipose PROT AUCs*

|  |  |  | ADD | | | | |
| --- | --- | --- | --- | --- | --- | --- | --- |
|  |  |  | 0 | 31.7 | 82.8 | 179.9 | 300.4 |
|  |  | Time point (days) | 0 | 2 | 6 | 14 | 24 |
| ADD | 0 | 0 |  |  |  |  |  |
|  | 31.7 | 2 | 🗸 |  |  |  |  |
|  | 82.8 | 6 | 🗴 | 🗸 |  |  |  |
|  | 179.9 | 14 | 🗸 | 🗸 | 🗸 |  |  |
|  | 300.4 | 24 | 🗸 | 🗸 | 🗸 | 🗸 |  |

🗸: significant (p < 0.05) relationship present; 🗴: significant (p < 0.05) relationship absent

Table S17. *Relationship significance between time points for F adipose PROT AUCs*

|  |  |  | ADD | | |
| --- | --- | --- | --- | --- | --- |
|  |  |  | 0 | 196.9 | 340.4 |
|  |  | Time point (days) | 0 | 14 | 24 |
| ADD | 0 | 0 |  |  |  |
|  | 196.9 | 14 | 🗸 |  |  |
|  | 340.4 | 24 | 🗸 | 🗴 |  |

🗸: significant (p < 0.05) relationship present; 🗴: significant (p < 0.05) relationship absent


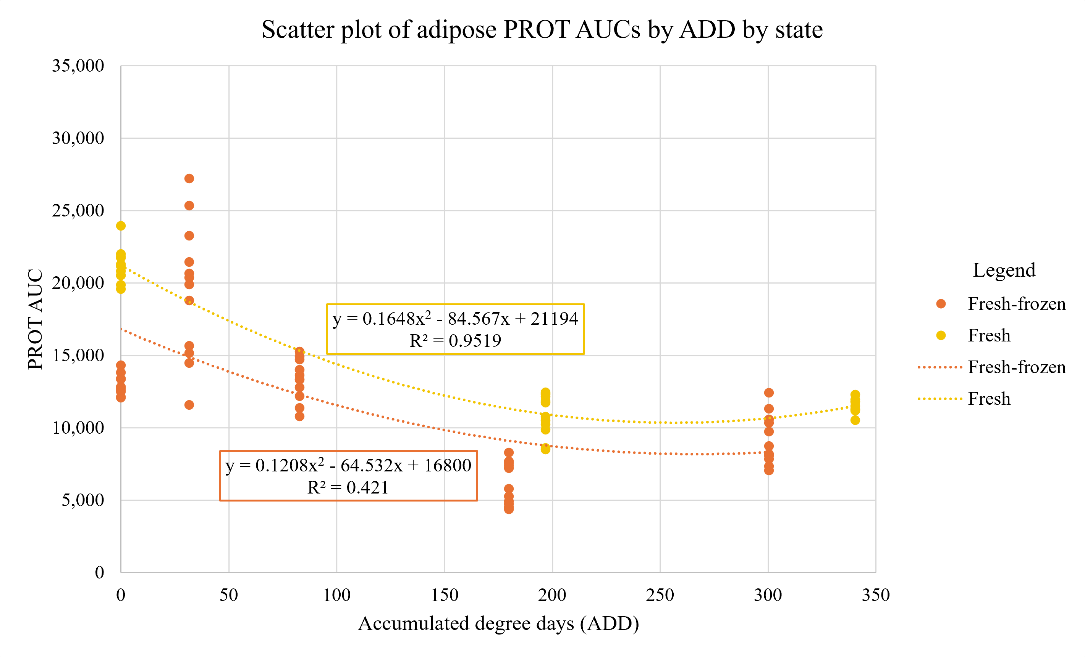


*Figure S13.* Scatter plot of adipose PROT AUCs by ADD by state

Table S18. *Relationship significance between time points for FFR adipose FOX AUCs*

|  |  |  | ADD | | | | |
| --- | --- | --- | --- | --- | --- | --- | --- |
|  |  |  | 0 | 31.7 | 82.8 | 179.9 | 300.4 |
|  |  | Time point (days) | 0 | 2 | 6 | 14 | 24 |
| ADD | 0 | 0 |  |  |  |  |  |
|  | 31.7 | 2 | 🗴 |  |  |  |  |
|  | 82.8 | 6 | 🗴 | 🗴 |  |  |  |
|  | 179.9 | 14 | 🗸 | 🗸 | 🗸 |  |  |
|  | 300.4 | 24 | 🗸 | 🗸 | 🗸 | 🗸 |  |

🗸: significant (p < 0.05) relationship present; 🗴: significant (p < 0.05) relationship absent

Table S19. *Relationship significance between time points for F adipose FOX AUCs*

|  |  |  | ADD | | |
| --- | --- | --- | --- | --- | --- |
|  |  |  | 0 | 196.9 | 340.4 |
|  |  | Time point (days) | 0 | 14 | 24 |
| ADD | 0 | 0 |  |  |  |
|  | 196.9 | 14 | 🗴 |  |  |
|  | 340.4 | 24 | 🗸 | 🗸 |  |

🗸: significant (p < 0.05) relationship present; 🗴: significant (p < 0.05) relationship absent


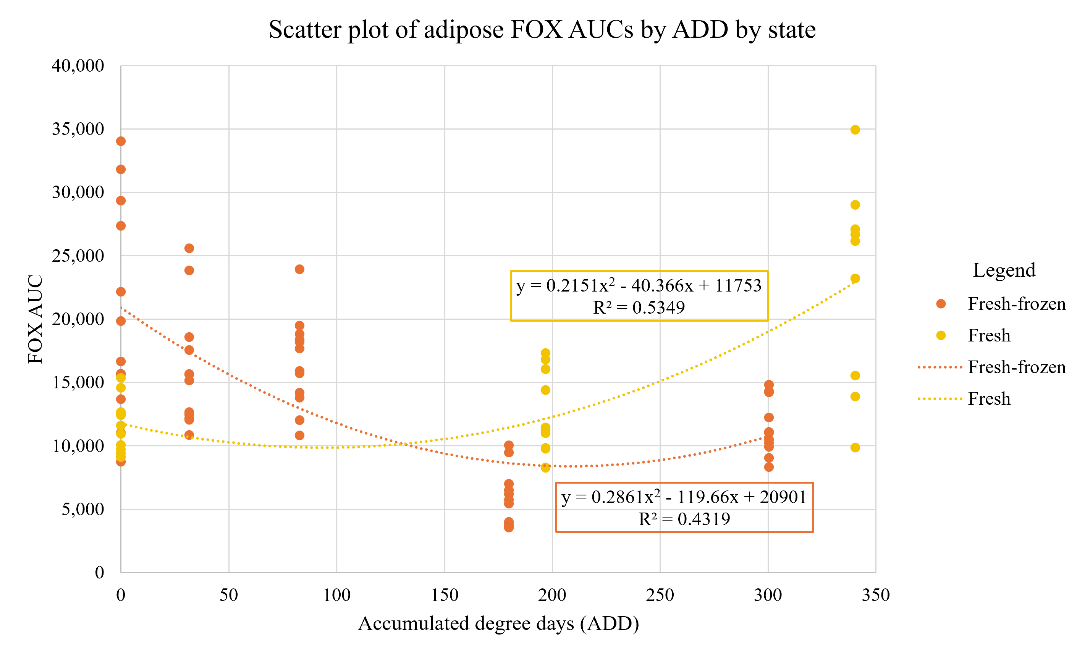


*Figure S14.* Scatter plot of adipose FOX AUCs by ADD by state

Table S20. *Relationship significance between time points for FFR adipose PROT-FOX ratios*

|  |  |  | ADD | | | | |
| --- | --- | --- | --- | --- | --- | --- | --- |
|  |  |  | 0 | 31.7 | 82.8 | 179.9 | 300.4 |
|  |  | Time point (days) | 0 | 2 | 6 | 14 | 24 |
| ADD | 0 | 0 |  |  |  |  |  |
|  | 31.7 | 2 | 🗸 |  |  |  |  |
|  | 82.8 | 6 | 🗴 | 🗸 |  |  |  |
|  | 179.9 | 14 | 🗸 | 🗴 | 🗴 |  |  |
|  | 300.4 | 24 | 🗴 | 🗸 | 🗴 | 🗴 |  |

🗸: significant (p < 0.05) relationship present; 🗴: significant (p < 0.05) relationship absent

Table S21. *Relationship significance between time points for F adipose PROT-FOX ratios*

|  |  |  | ADD | | |
| --- | --- | --- | --- | --- | --- |
|  |  |  | 0 | 196.9 | 340.4 |
|  |  | Time point (days) | 0 | 14 | 24 |
| ADD | 0 | 0 |  |  |  |
|  | 196.9 | 14 | 🗸 |  |  |
|  | 340.4 | 24 | 🗸 | 🗸 |  |

🗸: significant (p < 0.05) relationship present; 🗴: significant (p < 0.05) relationship absent


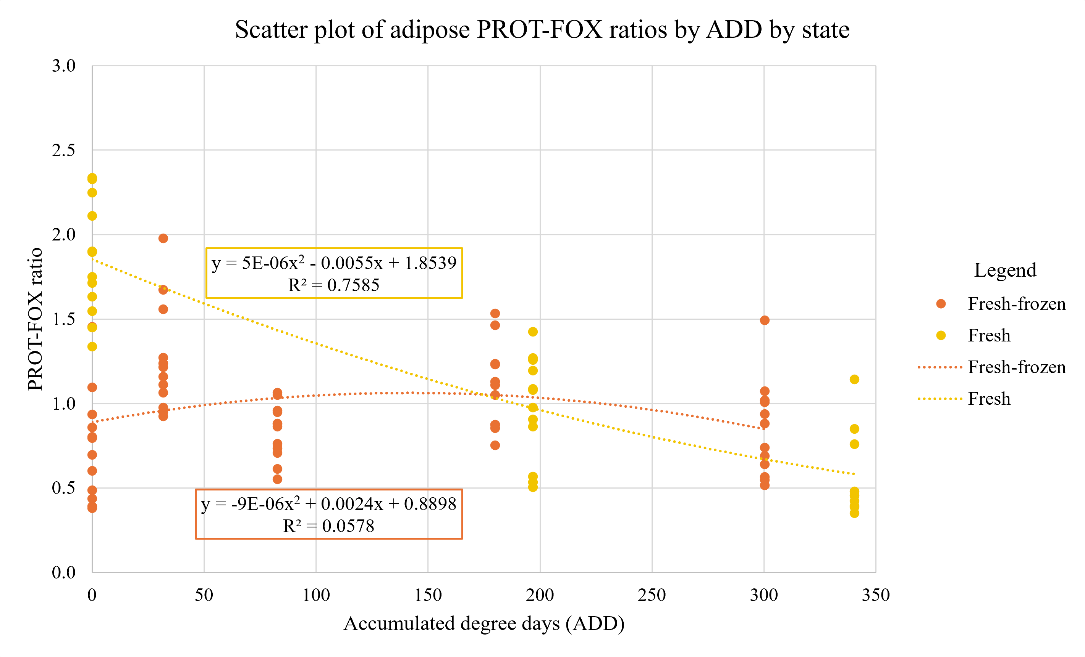


*Figure S15.* Scatter plot of adipose PROT-FOX ratios by ADD by state

*Muscle*

Table S22. *Relationship significance between time points for FFR muscle PROT AUCs*

|  |  |  | ADD | | | | |
| --- | --- | --- | --- | --- | --- | --- | --- |
|  |  |  | 0 | 31.7 | 82.8 | 179.9 | 300.4 |
|  |  | Time point (days) | 0 | 2 | 6 | 14 | 24 |
| ADD | 0 | 0 |  |  |  |  |  |
|  | 31.7 | 2 | 🗴 |  |  |  |  |
|  | 82.8 | 6 | 🗴 | 🗴 |  |  |  |
|  | 179.9 | 14 | 🗴 | 🗴 | 🗴 |  |  |
|  | 300.4 | 24 | 🗸 | 🗸 | 🗸 | 🗸 |  |

🗸: significant (p < 0.05) relationship present; 🗴: significant (p < 0.05) relationship absent

Table S23. *Relationship significance between time points for F muscle PROT AUCs*

|  |  |  | ADD | | |
| --- | --- | --- | --- | --- | --- |
|  |  |  | 0 | 196.9 | 340.4 |
|  |  | Time point (days) | 0 | 14 | 24 |
| ADD | 0 | 0 |  |  |  |
|  | 196.9 | 14 | 🗸 |  |  |
|  | 340.4 | 24 | 🗸 | 🗸 |  |

🗸: significant (p < 0.05) relationship present; 🗴: significant (p < 0.05) relationship absent

*
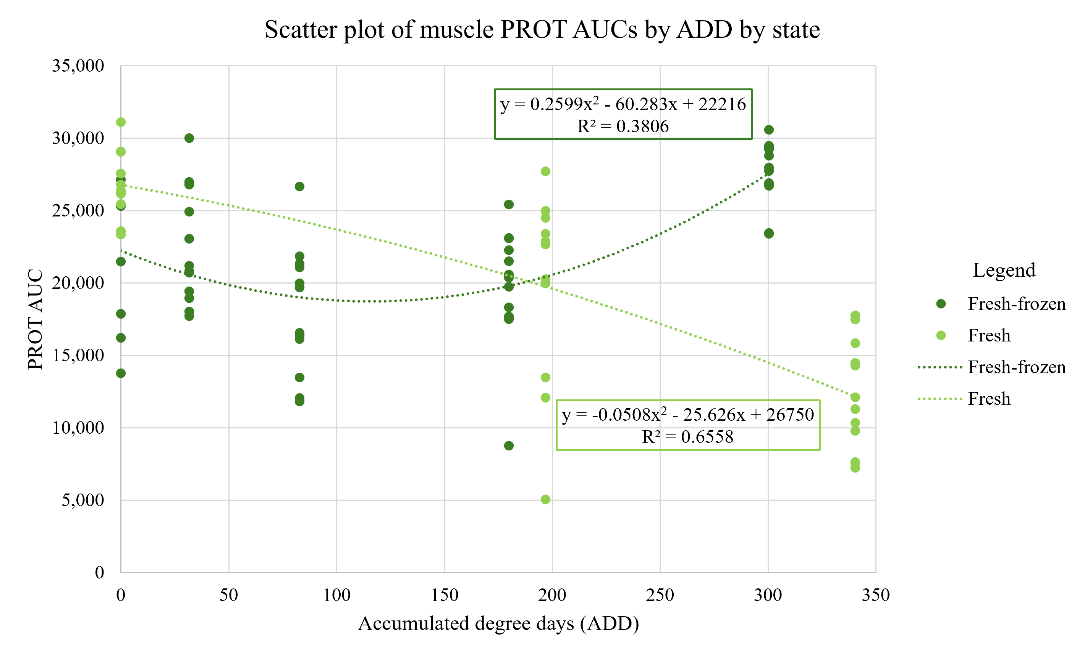
*

*Figure S16.* Scatter plot of muscle PROT AUCs by ADD by state

Table S24. *Relationship significance between time points for FFR muscle FOX AUCs*

|  |  |  | ADD | | | | |
| --- | --- | --- | --- | --- | --- | --- | --- |
|  |  |  | 0 | 31.7 | 82.8 | 179.9 | 300.4 |
|  |  | Time point (days) | 0 | 2 | 6 | 14 | 24 |
| ADD | 0 | 0 |  |  |  |  |  |
|  | 31.7 | 2 | 🗸 |  |  |  |  |
|  | 82.8 | 6 | 🗴 | 🗸 |  |  |  |
|  | 179.9 | 14 | 🗴 | 🗴 | 🗴 |  |  |
|  | 300.4 | 24 | 🗴 | 🗴 | 🗸 | 🗴 |  |

🗸: significant (p < 0.05) relationship present; 🗴: significant (p < 0.05) relationship absent

Table S25. *Relationship significance between time points for F muscle FOX AUCs*

|  |  |  | ADD | | |
| --- | --- | --- | --- | --- | --- |
|  |  |  | 0 | 196.9 | 340.4 |
|  |  | Time point (days) | 0 | 14 | 24 |
| ADD | 0 | 0 |  |  |  |
|  | 196.9 | 14 | 🗴 |  |  |
|  | 340.4 | 24 | 🗸 | 🗸 |  |

🗸: significant (p < 0.05) relationship present; 🗴: significant (p < 0.05) relationship absent


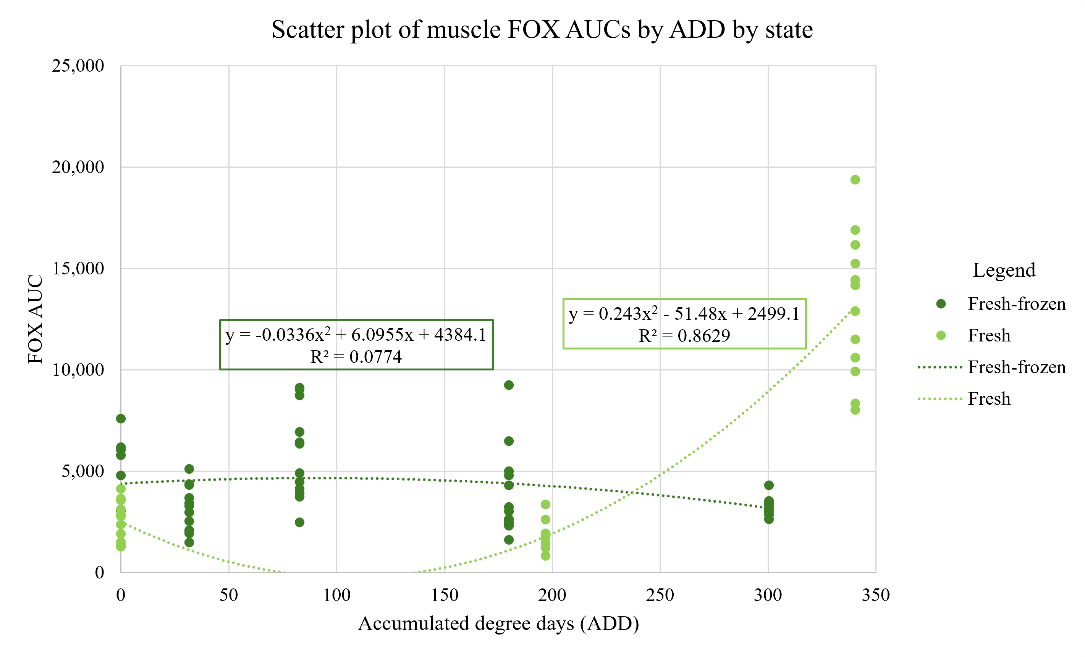


*Figure S17.* Scatter plot of muscle FOX AUCs by ADD by state

Table S26. *Relationship significance between time points for FFR muscle PROT-FOX ratios*

|  |  |  | ADD | | | | |
| --- | --- | --- | --- | --- | --- | --- | --- |
|  |  |  | 0 | 31.7 | 82.8 | 179.9 | 300.4 |
|  |  | Time point (days) | 0 | 2 | 6 | 14 | 24 |
| ADD | 0 | 0 |  |  |  |  |  |
|  | 31.7 | 2 | 🗸 |  |  |  |  |
|  | 82.8 | 6 | 🗴 | 🗸 |  |  |  |
|  | 179.9 | 14 | 🗴 | 🗴 | 🗴 |  |  |
|  | 300.4 | 24 | 🗸 | 🗴 | 🗸 | 🗴 |  |

🗸: significant (p < 0.05) relationship present; 🗴: significant (p < 0.05) relationship absent

Table S27. *Relationship significance between time points for F muscle PROT-FOX ratios*

|  |  |  | ADD | | |
| --- | --- | --- | --- | --- | --- |
|  |  |  | 0 | 196.9 | 340.4 |
|  |  | Time point (days) | 0 | 14 | 24 |
| ADD | 0 | 0 |  |  |  |
|  | 196.9 | 14 | 🗴 |  |  |
|  | 340.4 | 24 | 🗸 | 🗸 |  |

🗸: significant (p < 0.05) relationship present; 🗴: significant (p < 0.05) relationship absent

**
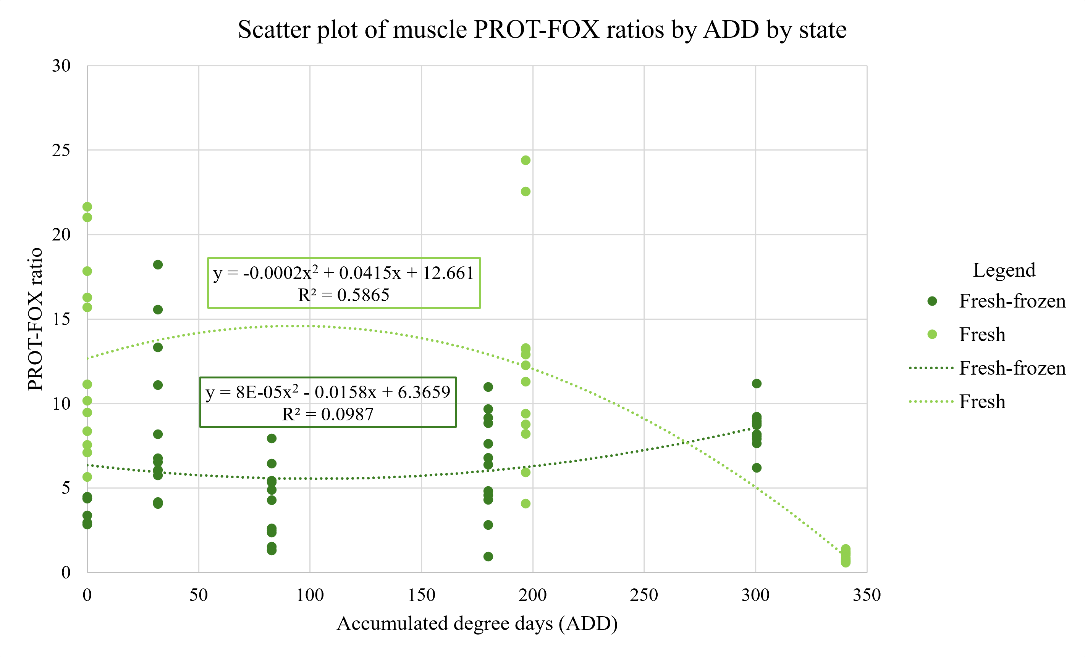
**

*Figure S18.* Scatter plot of muscle PROT-FOX ratios by ADD by state

**N: Occurrence of significant (p < 0.05) relationships between fresh and fresh-frozen samples at corresponding time points by tissue type**

Table S28. *Occurrence of significant (p < 0.05) relationships between F and FFR samples at corresponding time points per tissue type*

|  |  | **Occurrence of significant (p < 0.05) relationship between F and FFR parameters** | | |
| --- | --- | --- | --- | --- |
| **Tissue type** | **Time point (days)** | **PROT AUCs** | **FOX AUCs** | **PROT-FOX ratios** |
| Skin | 0 | 🗸 | 🗴 | 🗸 |
|  | 14 | 🗴 | 🗴 | 🗴 |
|  | 24 | 🗸 | 🗸 | 🗸 |
| Adipose | 0 | 🗸 | 🗸 | 🗸 |
|  | 14 | 🗸 | 🗸 | 🗴 |
|  | 24 | 🗸 | 🗸 | 🗸 |
| Muscle | 0 | 🗸 | 🗸 | 🗸 |
|  | 14 | 🗴 | 🗸 | 🗸 |
|  | 24 | 🗸 | 🗸 | 🗸 |

🗸: significant (p < 0.05) relationship present; 🗴: significant (p < 0.05) relationship absent

**O: EEM peaks excluded from fluorophore peak analysis**

Table S29. *Overview of fluorophore peaks excluded from fluorophore peak analysis*

|  |  | **Occurrence of peak** | | |
| --- | --- | --- | --- | --- |
| **Name assigned to peak** | **Central peak coordinates (excitation/emission wavelengths in nm)** | **Tissue type** | **Treatment group** | **Time point (days)** |
| Unknown A | 425/592 | Adipose | FFR | 24 |
| Unknown A | 430/595.5 | Muscle | FFR | 6 |
| Unknown B | 355/544 | Adipose | FFR | 24 |
| Unknown C | 545/585.5 | Adipose | F | 0 |
| Unknown C | 555/595 | Muscle | FFR | 6 |
| Unknown D | 260/375.5 | Adipose | F | 14 |
| Unknown E | 295/501.5 | Skin | F | 0 |
| Unknown F | 240/333.5 | Muscle | FFR | 14 |

**P: Occurrence of fluorophore peaks**

Table S30. *Overview fluorophore peak occurrences in skin EEMs*

|  |  |  |  | **Occurrence of peak** | | | | |
| --- | --- | --- | --- | --- | --- | --- | --- | --- |
| **Tissue type** | **Treatment group** | **ADD** | **Time point (days)** | **Peak A** | **Peak B** | **Peak C** | **Peak D** | **Peak E** |
| Skin | FFR | 0 | 0 | 🗸 | 🗴 | 🗸 | 🗴 | 🗴 |
|  | FFR | 31.7 | 2 | 🗸 | 🗴 | 🗸 | 🗴 | 🗴 |
|  | FFR | 82.8 | 6 | 🗸 | 🗴 | 🗸 | 🗸 | 🗸 |
|  | FFR | 179.9 | 14 | 🗸 | 🗸 | 🗴 | 🗸 | 🗴 |
|  | FFR | 300.4 | 24 | 🗸 | 🗴 | 🗸 | 🗴 | 🗸 |
|  | F | 0 | 0 | 🗸 | 🗴 | 🗸 | 🗴 | 🗸 |
|  | F | 196.9 | 14 | 🗸 | 🗴 | 🗸 | 🗴 | 🗸 |
|  | F | 340.4 | 24 | 🗸 | 🗸 | 🗸 | 🗴 | 🗴 |

🗸: peak present; 🗴: peak absent

Table S31. *Overview fluorophore peak occurrences in adipose EEMs*

|  | | | | **Occurrence of peak** | | | | |
| --- | --- | --- | --- | --- | --- | --- | --- | --- |
| **Tissue type** | **Treatment group** | **ADD** | **Time point (days)** | **Peak A** | **Peak B** | **Peak C** | **Peak D** | **Peak E** |
| Adipose | FFR | 0 | 0 | 🗸 | 🗸 | 🗸 | 🗸 | 🗴 |
|  | FFR | 31.7 | 2 | 🗸 | 🗸 | 🗴 | 🗸 | 🗴 |
|  | FFR | 82.8 | 6 | 🗸 | 🗸 | 🗸 | 🗴 | 🗴 |
|  | FFR | 179.9 | 14 | 🗸 | 🗸 | 🗸 | 🗸 | 🗸 |
|  | FFR | 300.4 | 24 | 🗸 | 🗸 | 🗸 | 🗴 | 🗸 |
|  | F | 0 | 0 | 🗸 | 🗸 | 🗴 | 🗴 | 🗸 |
|  | F | 196.9 | 14 | 🗴 | 🗸 | 🗴 | 🗸 | 🗸 |
|  | F | 340.4 | 24 | 🗸 | 🗸 | 🗴 | 🗴 | 🗸 |

🗸: peak present; 🗴: peak absent

Table S32. *Overview fluorophore peak occurrences in muscle EEMs*

|  |  |  |  | **Occurrence of peak** | | | | |
| --- | --- | --- | --- | --- | --- | --- | --- | --- |
| **Tissue type** | **Treatment group** | **ADD** | **Time point (days)** | **Peak A** | **Peak B** | **Peak C** | **Peak D** | **Peak E** |
| Muscle | FFR | 0 | 0 | 🗸 | 🗴 | 🗴 | 🗴 | 🗸 |
|  | FFR | 31.7 | 2 | 🗸 | 🗴 | 🗴 | 🗴 | 🗴 |
|  | FFR | 82.8 | 6 | 🗸 | 🗸 | 🗸 | 🗸 | 🗴 |
|  | FFR | 179.9 | 14 | 🗸 | 🗴 | 🗴 | 🗴 | 🗴 |
|  | FFR | 300.4 | 24 | 🗸 | 🗸 | 🗴 | 🗴 | 🗴 |
|  | F | 0 | 0 | 🗸 | 🗴 | 🗴 | 🗴 | 🗴 |
|  | F | 196.9 | 14 | 🗸 | 🗴 | 🗴 | 🗴 | 🗴 |
|  | F | 340.4 | 24 | 🗸 | 🗸 | 🗸 | 🗸 | 🗸 |

🗸: peak present; 🗴: peak absent

**Q: Effect of multiple freeze-thaw cycles on PROT and FOX fluorescence in time point 2 samples**

The ratio of one to two F-T cycles was calculated for each fluorescence parameter (PROT and FOX AUCs, and PROT-FOX ratios) by dividing the fluorescence intensity at each emission wavelength after one F-T cycle, by the values obtained after the second cycle. If no changes in fluorescence occur from one to two cycles, the ratio is expected to be 1, and if there is a change, the greater this change, the further away from 1 the ratio will be.


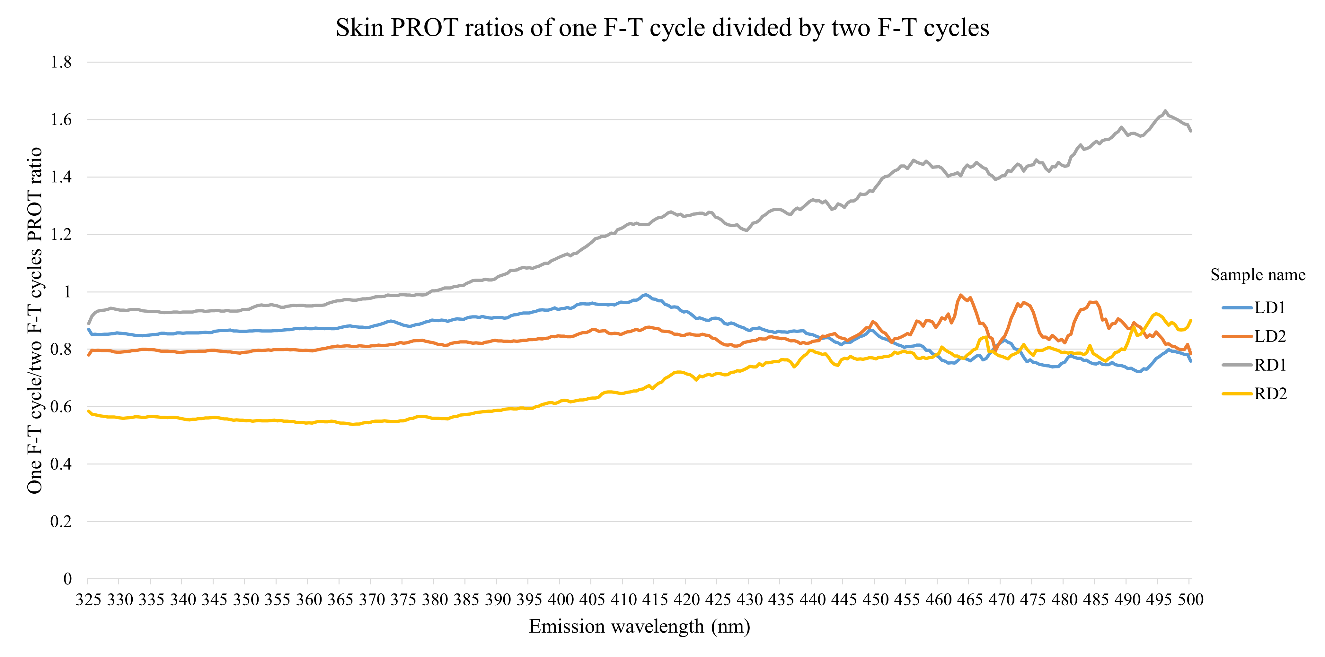


*Figure S19.* Skin time point 2 PROT ratios following one vs. two freeze-thaw cycles


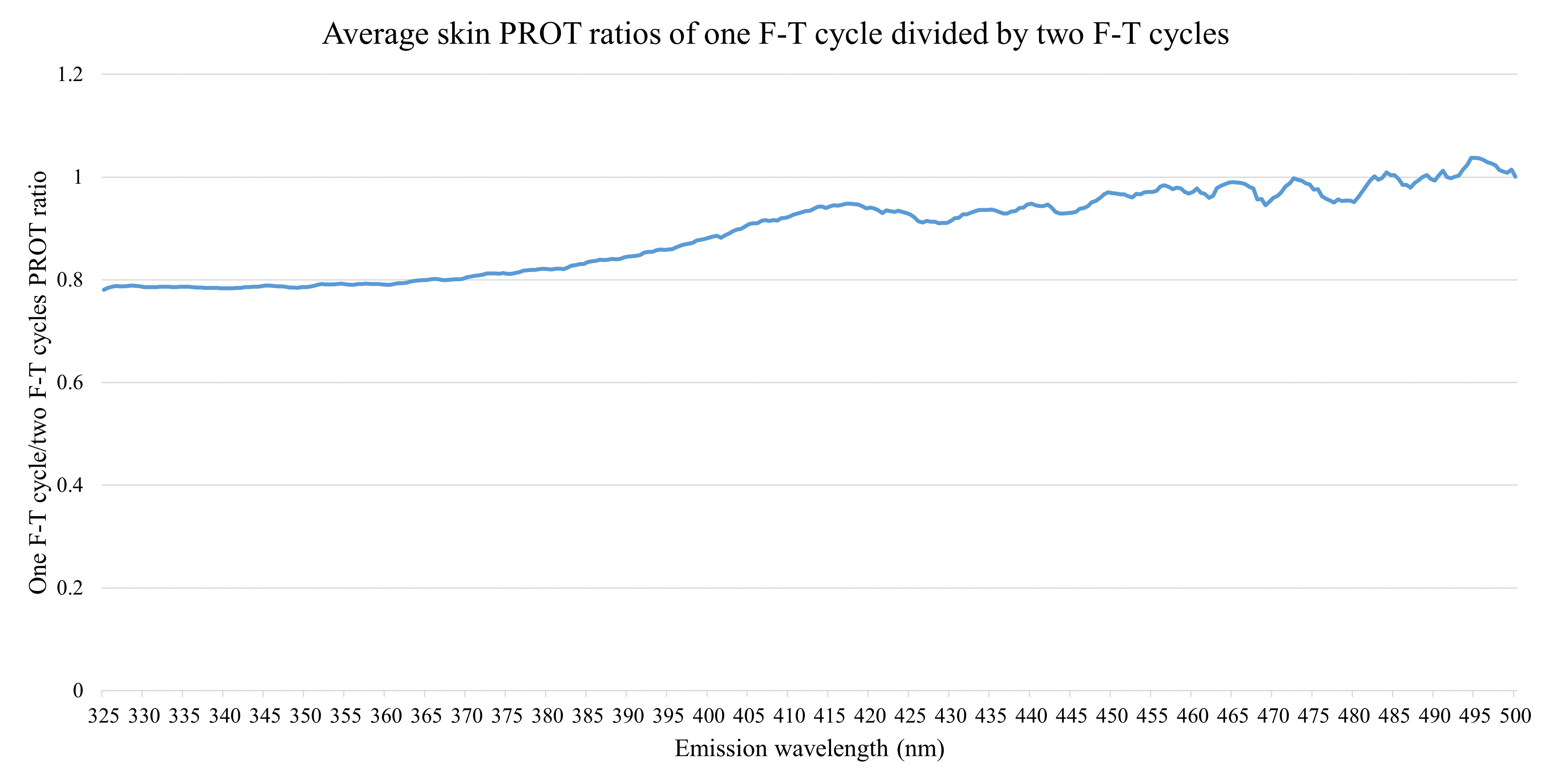


*Figure S20.* Average skin time point 2 PROT ratios following one vs. two freeze-thaw cycles


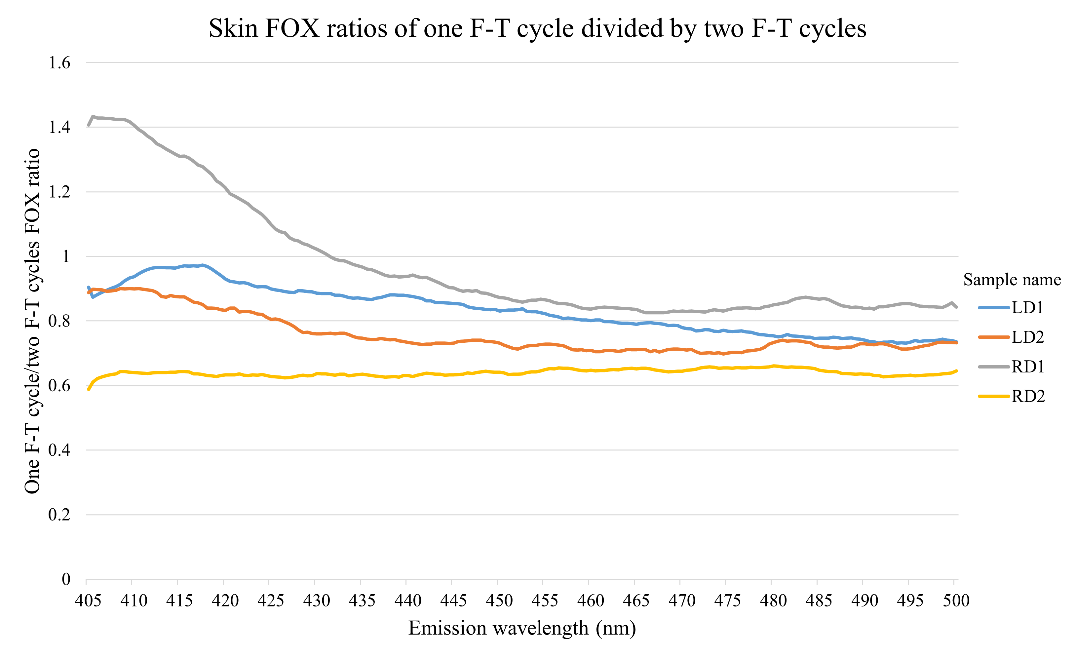


*Figure S21.* Skin time point 2 FOX ratios following one vs. two freeze-thaw cycles


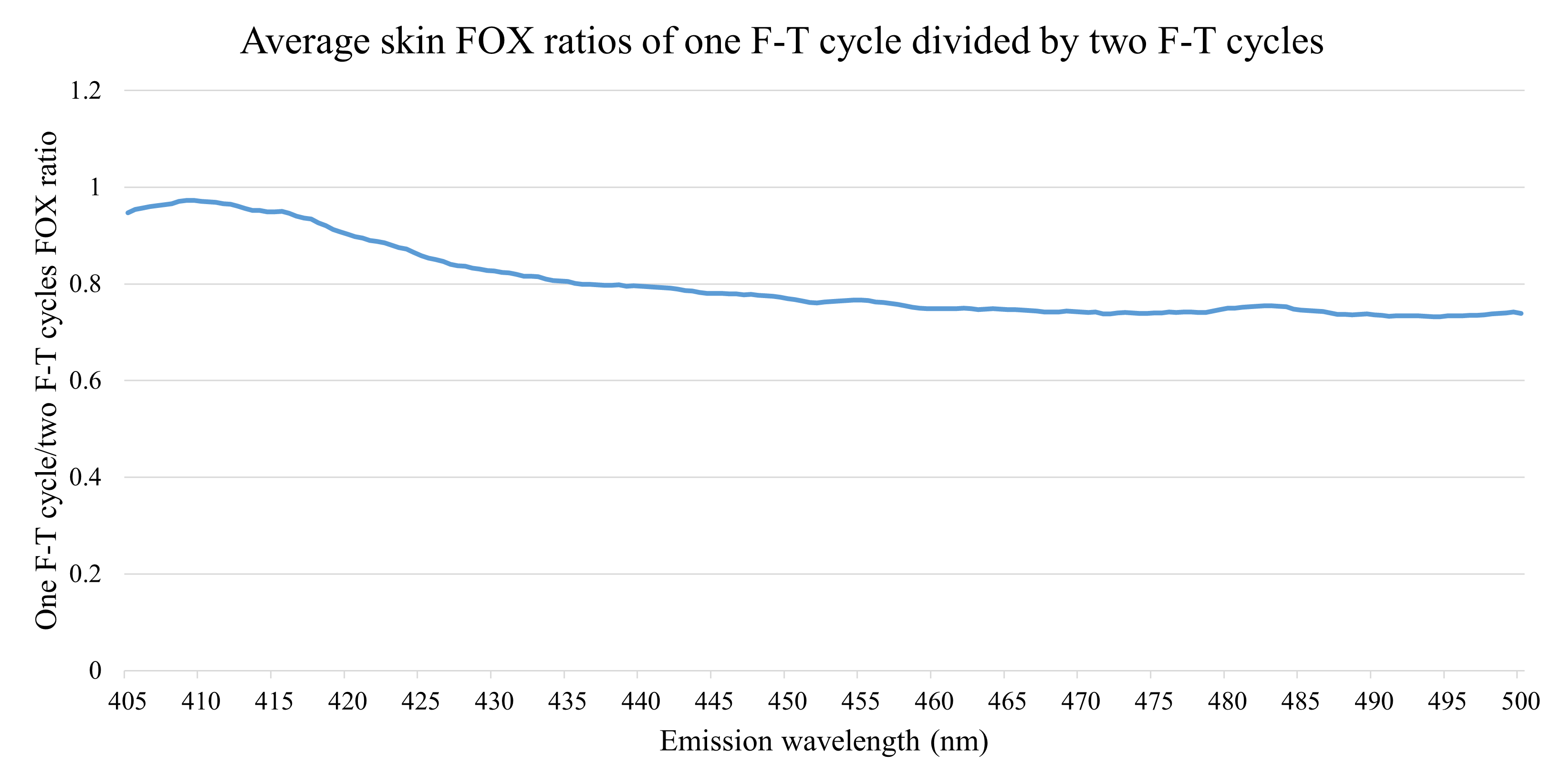


*Figure S22.* Average skin time point 2 FOX ratios following one vs. two freeze-thaw cycles


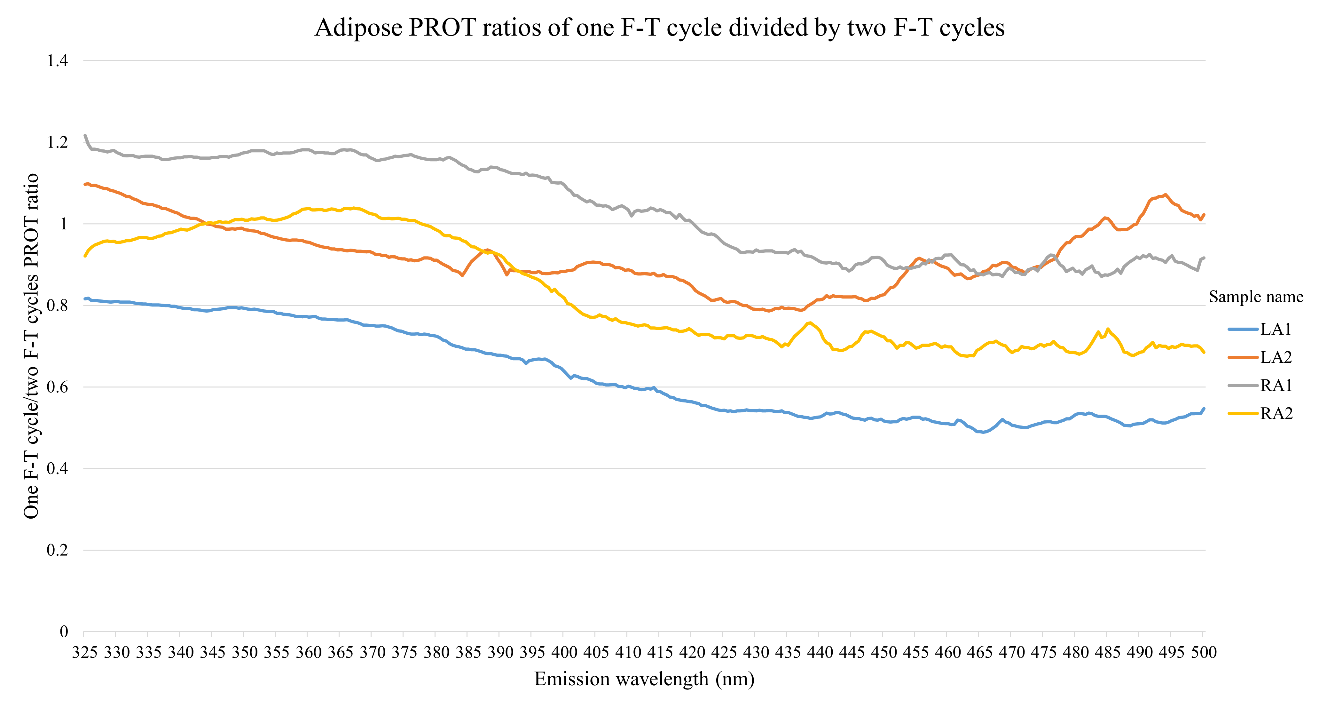


*Figure S23.* Adipose time point 2 PROT ratios following one vs. two freeze-thaw cycles


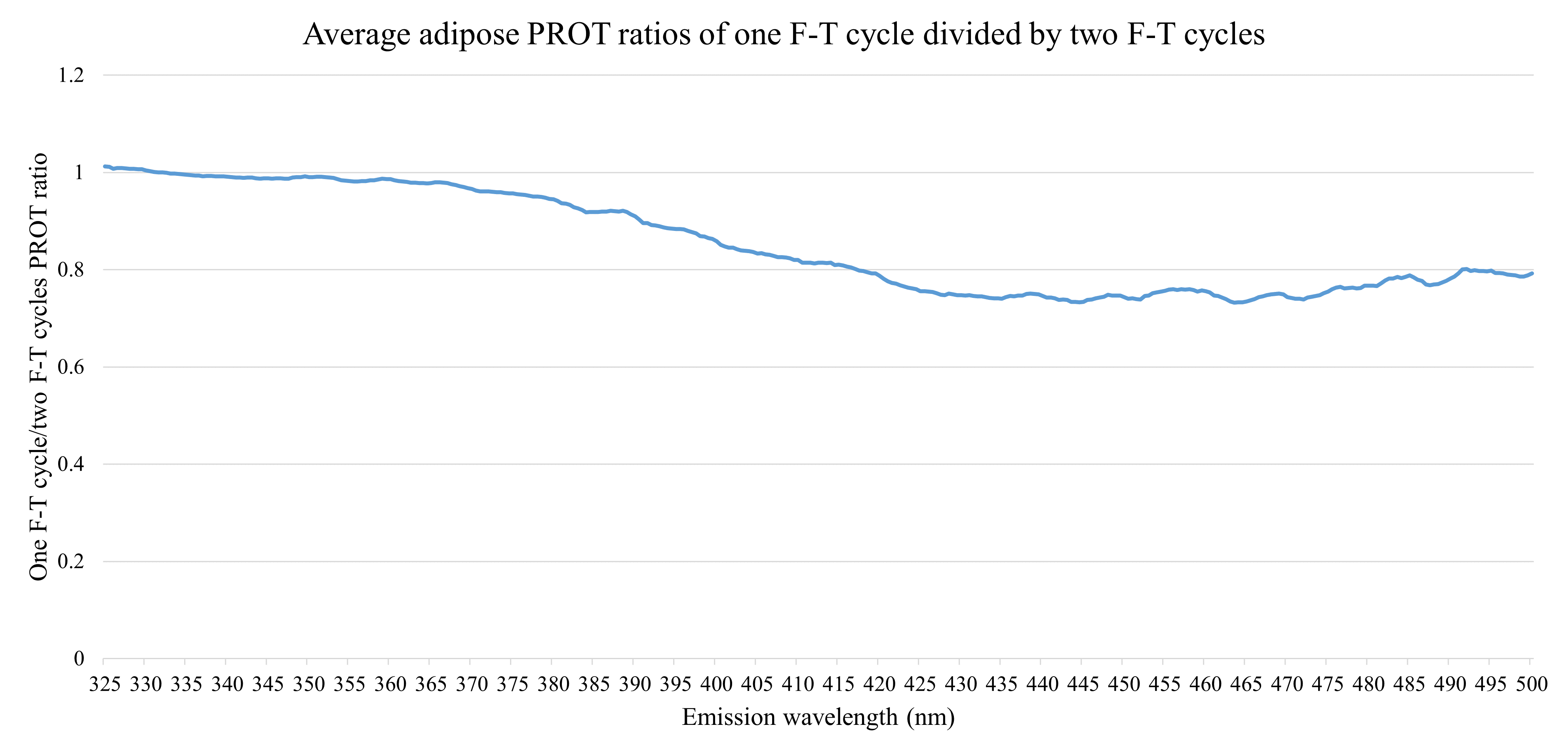


*Figure S24.* Average adipose time point 2 PROT ratios following one vs. two freeze-thaw cycles


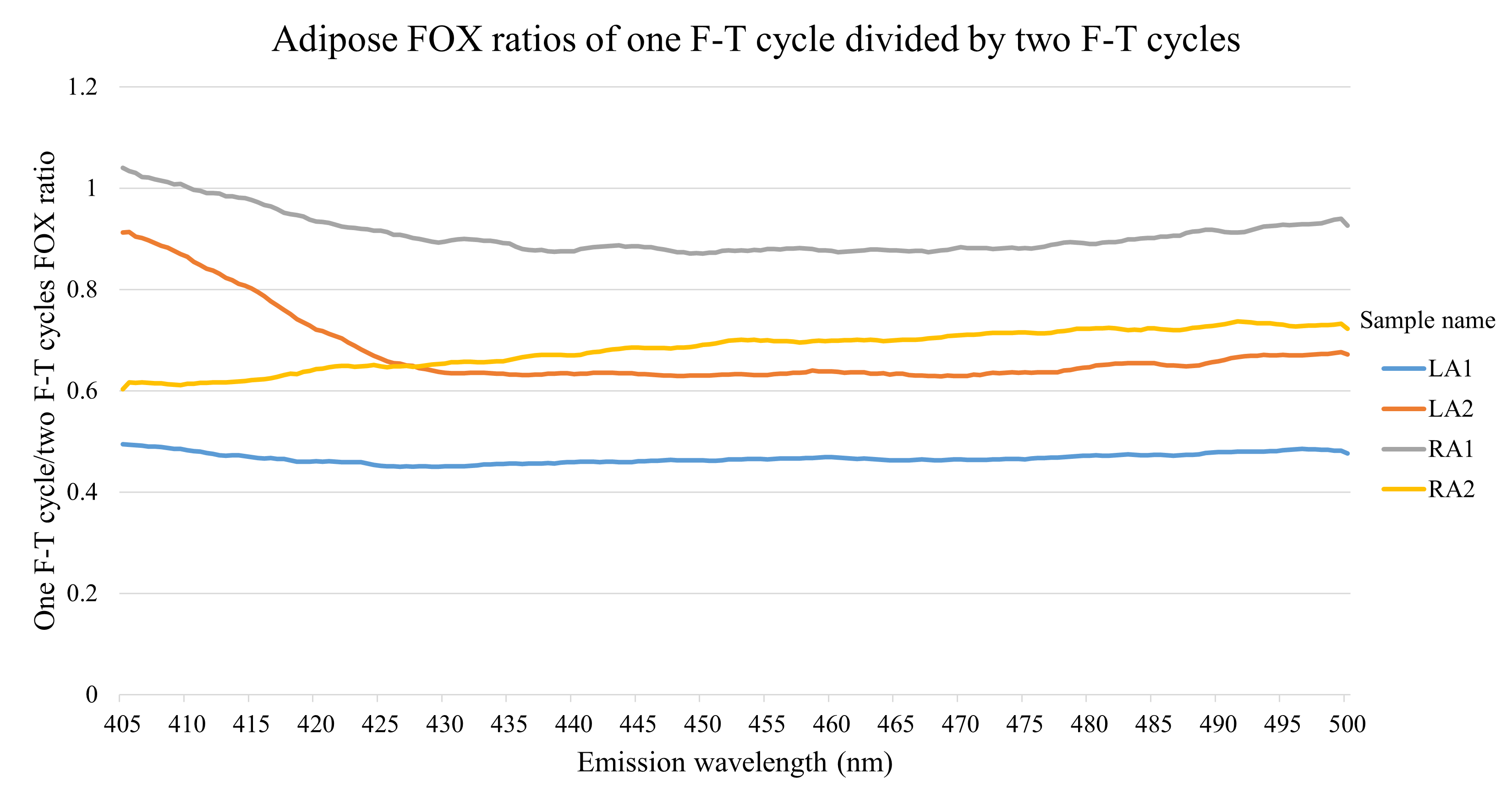


*Figure S25.* Adipose time point 2 FOX ratios following one vs. two freeze-thaw cycles


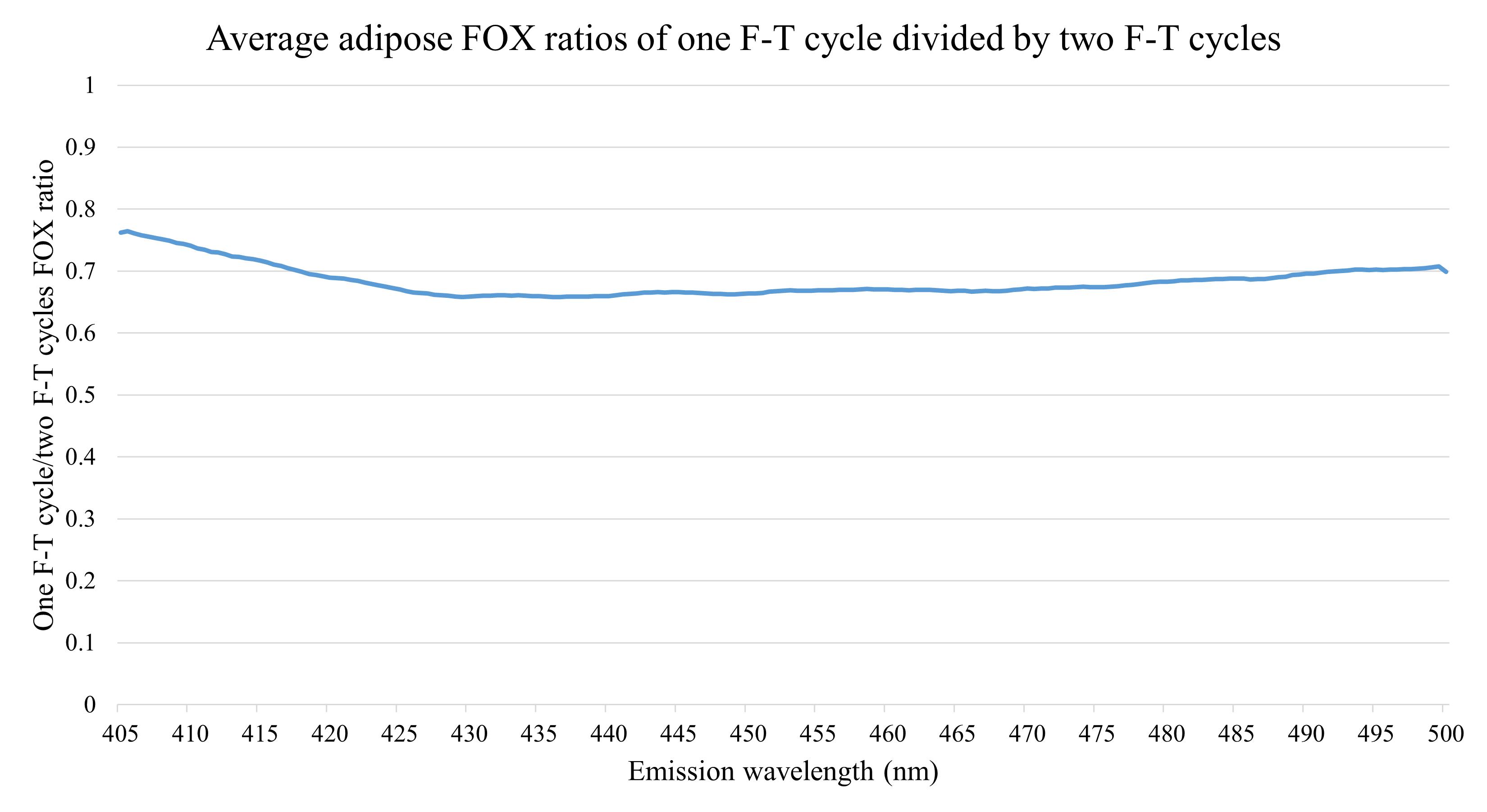


*Figure S26.* Average adipose time point 2 FOX ratios following one vs. two freeze-thaw cycles


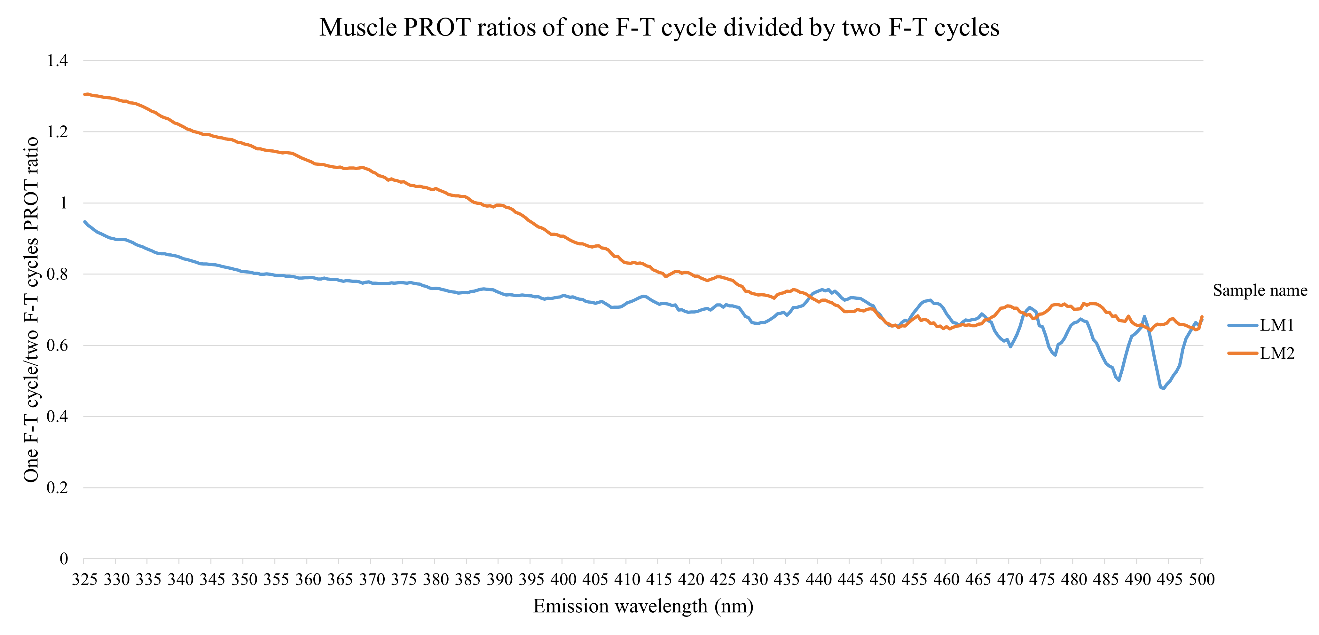


*Figure S27.* Muscle time point 2 PROT ratios following one vs. two freeze-thaw cycles


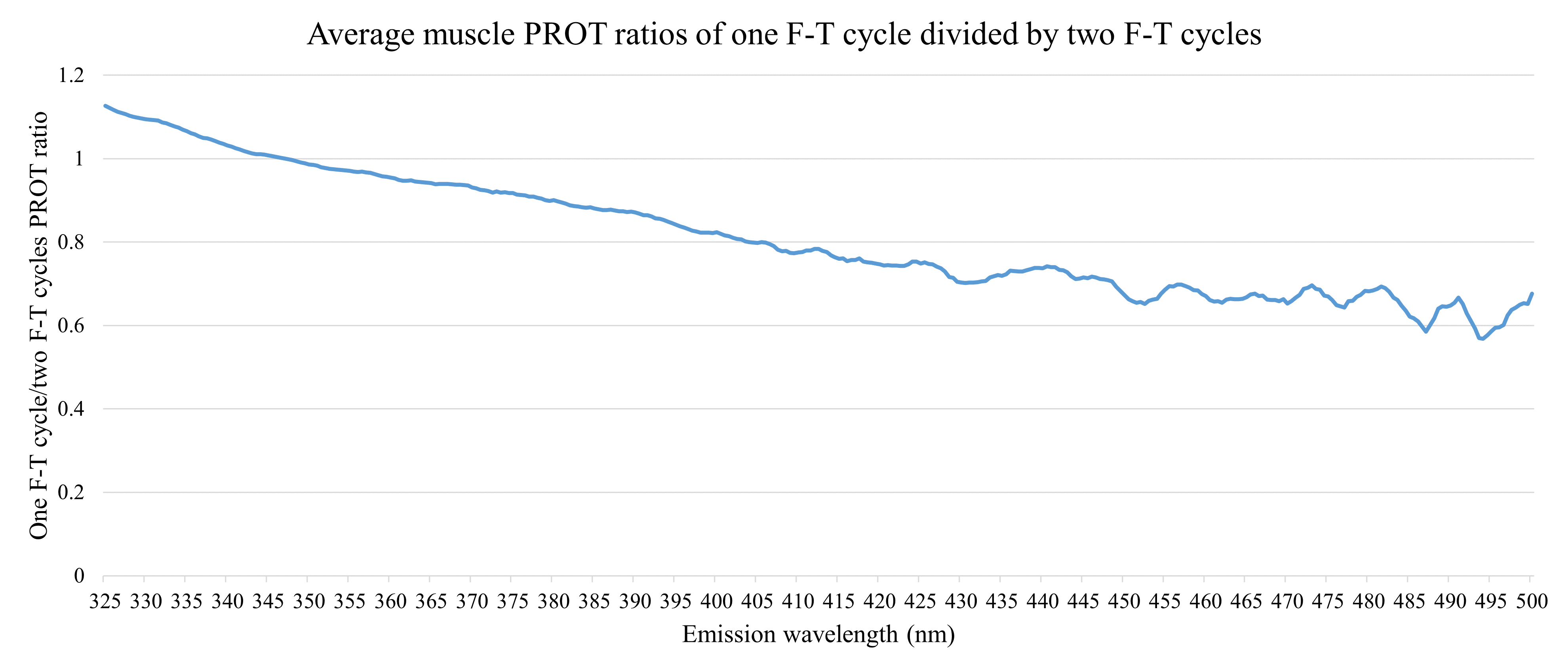


*Figure S28.* Average muscle time point 2 PROT ratios following one vs. two freeze-thaw cycles


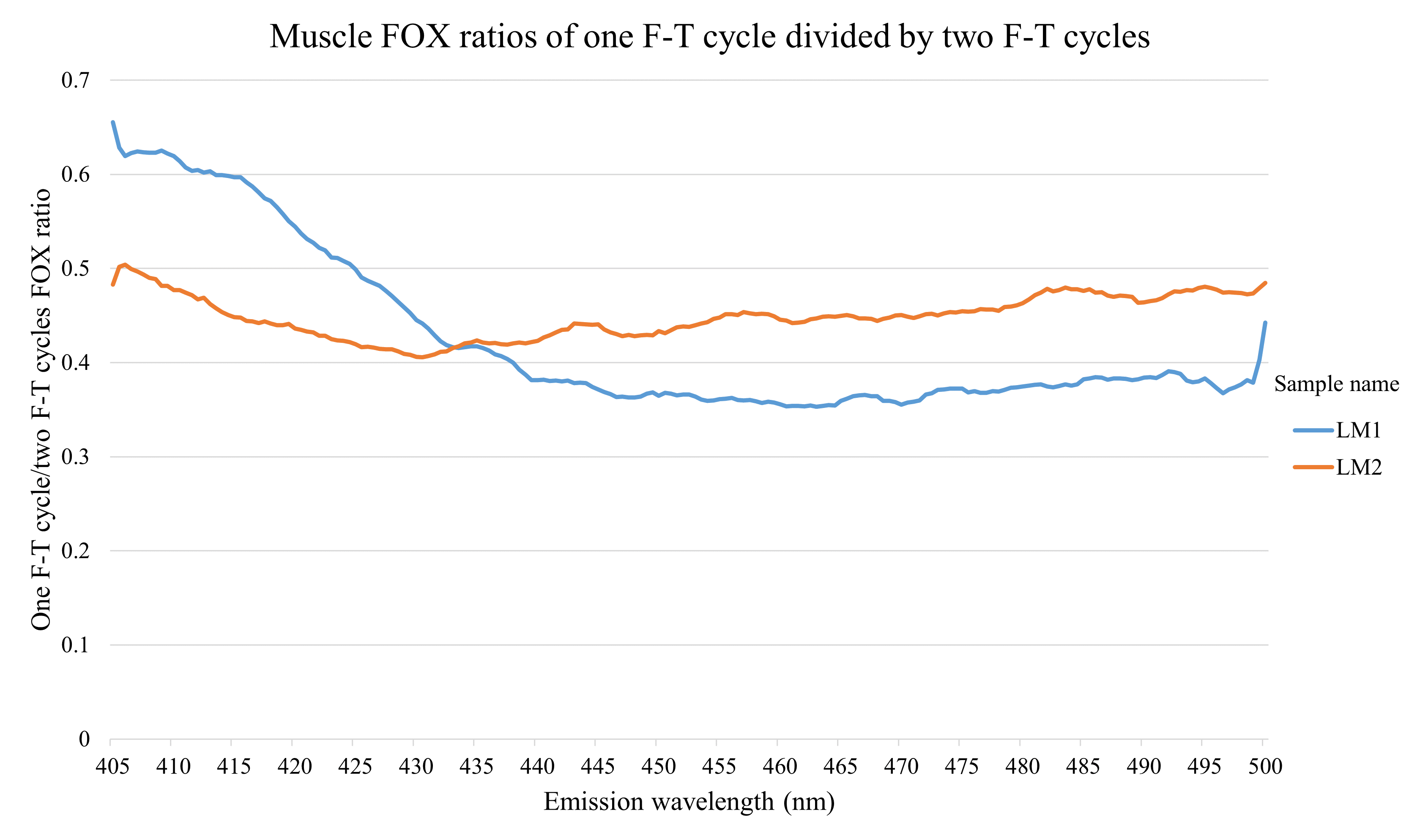


*Figure S29.* Muscle FOX ratios following one vs. two freeze-thaw cycles


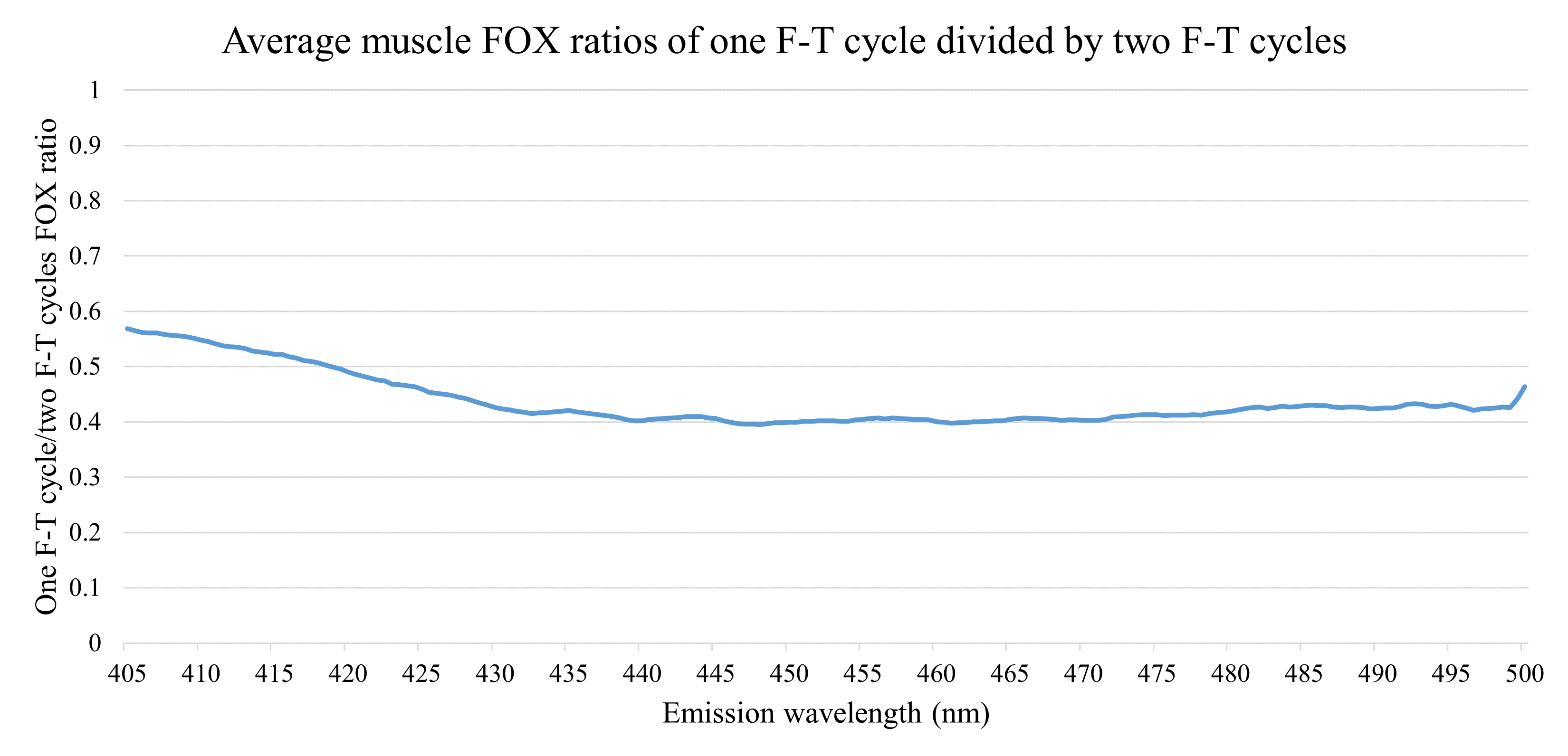


*Figure S30.* Average muscle time point 2 FOX ratios following one vs. two freeze-thaw cycles

# References

1. Weather archive in Amsterdam (airport). (n.d.). Reliable Prognosis. Retrieved 25 October 2022, from https://rp5.ru/Weather_archive_in_Amsterdam_(airport)

2. Lameira, A. P., Gawryszewski, L. G., Silva, S. G., Ferreira, F. M., Vargas, C., Umiltà, C., & Pereira, A. (2009). *Hand posture effects on handedness recognition as revealed by the Simon effect*. *3*, 1–8.

3. van Dam, A. (2022, September 21). [Personal communication].
